# Systematic evaluation of phenotypic variations induced by prophages in a clinical isolate of *Pseudomonas aeruginosa*

**DOI:** 10.1128/msystems.00428-25

**Published:** 2025-08-15

**Authors:** Xiaoyu Li, Yang Tao, Bingrui Sui, DanDan Li, Lili Wang, Yumin Hou, Yongping Xu, Bijie Hu, Na Li, Demeng Tan

**Affiliations:** 1 MOE Key Laboratory of Bio-Intelligent Manufacturing, School of Bioengineering, Dalian University of Technology 12399, Dalian, China; 2 Department of Infectious Disease, Zhongshan Hospital, Fudan University 92323, Shanghai, China; 3 Shanghai Public Health Clinical Center, Fudan University 12478, Shanghai, China; Universidad de Los Andes, Bogota, Colombia

**Keywords:** prophage, phage-host interactions, *Pseudomonas aeruginosa*, fitness, lysogen

## Abstract

With the rise of antibiotic resistance, nosocomial infections caused by *Pseudomonas aeruginosa* present a significant challenge to healthcare systems. Prophages integrated into bacterial chromosomes play a key role in generating phenotypic and genotypic diversity, influencing bacterial pathogenicity and complicating antimicrobial treatment strategies. Recent advances in high-throughput sequencing have facilitated the precise localization of prophages within bacterial genomes. However, the mechanisms by which prophages influence host phenotypes remain poorly understood, particularly in clinical polylysogens. In this study, we conducted a comprehensive investigation of prophages predicted in the clinical *P. aeruginosa* isolate ZS-PA-05 in the absence of an external trigger, focusing on their contribution to bacterial phenotype through the use of prophage deletion mutants. Our findings indicate that ZS-PA-05 harbors both active and cryptic prophages, each exhibiting unique spontaneous induction rates. Notably, the absence of certain prophages significantly affects bacterial growth, motility, biofilm formation, antibiotic susceptibility, and phage proliferation, as well as virulence. Intriguingly, the deletion of prophage Y significantly increased pyocyanin production, enhancing interspecies competition and cell line survival, despite impaired bacterial growth. This underscores the complexity of prophage–host interactions and emphasizes the role of prophages in bacterial adaptation, with far-reaching implications for antimicrobial therapies and beyond.

**IMPORTANCE:**

Upon infecting a bacterial host, phages can follow one of two developmental pathways: the lytic or lysogenic cycle. In the lysogenic state, prophages remain dormant, integrating into the bacterial genome and being vertically transmitted through binary fission. These prophages profoundly influence bacterial phenotypic and genetic diversity and contribute to the structuring of microbial communities. Here, we systematically assess the beneficial and detrimental impacts of prophage carriage in the clinical multilysogen *Pseudomonas aeruginosa* strain ZS-PA-05. Our results reveal marked variation in spontaneous induction frequencies among co-resident prophages and demonstrate prophage-driven phenotypic heterogeneity. By uncovering key aspects of prophage–host interactions, this study highlights the critical role of prophages in shaping the behavior of clinical isolates, particularly in the context of antimicrobial interventions such as antibiotic and phage therapies.

## INTRODUCTION

*Pseudomonas aeruginosa* is a ubiquitous gram-negative opportunistic pathogen and a growing public health threat (1). Frequently associated with healthcare settings, it causes a wide range of infections linked to medical devices, surgical procedures, and chronic respiratory conditions, particularly in immunocompromised individuals and patients with cystic fibrosis (2, 3). A defining feature of *P. aeruginosa* clinical isolates is their remarkable phenotypic diversity and intrinsic heterogeneity, driven by highly dynamic genomes that promote persistence and antibiotic resistance, posing significant challenges for treatment (4, 5). The advent of high-throughput short- and long-read sequencing technologies has enabled large-scale genomic analyses, revealing the extensive plasticity of the *P. aeruginosa* genome (6). These studies highlight the central role of horizontal gene transfer in shaping strain-specific genetic repertoires and driving the pathogen’s adaptability and resilience in diverse environments.

Mobile genetic elements, including transposons, integrons, plasmids, and phages, can integrate into bacterial chromosomes at specific loci or persist as extrachromosomal elements (7, 8). Early estimates indicate that prophages constitute 10%–20% of the bacterial genome (9). Their integration can provide a selective advantage to the host, particularly in the case of Pf phages, which significantly influence *Pseudomonas* virulence, modulate mammalian immune responses, and contribute to the persistence of chronic infections (10, 11). Recent studies in *P. aeruginosa* have demonstrated that filamentous phages are highly active in bacterial biofilms (12–14). In a murine pneumonia model, for instance, Pf prophage production by *P. aeruginosa* promoted biofilm formation, restricting bacterial dissemination from the lungs. Phage-driven mucin adhesion limited epithelial invasion, attenuated lung injury, reduced macrophage-mediated clearance, and conferred hallmark features of chronic infection absent in Pf-deficient strains (15). Thus, at a population level, bacteria would benefit from acquiring these inducible phages, and such acquisition could lead to rapidly changing phenotypic traits without incurring the physiological or metabolic fitness costs associated with the core genome.

Prophage research in *P. aeruginosa* has predominantly focused on inducible elements capable of excision and productive infection, while the functional impact of the broader prophage repertoire on host fitness across diverse environments remains poorly understood. Progressive genomic decay can trap prophages within the bacterial chromosome, driving the accumulation of mutations that impair excision, phage particle production, and reinfection, ultimately rendering them cryptic (16, 17). The phenotypic consequences of these degenerated or poorly inducible prophages have yet to be fully characterized. A study conducted by Rob Lavigne’s lab analyzing 241 *P*. *aeruginosa* strains revealed that Pf1-like genetic elements (Pf1, Pf4, and Pf5) are widely distributed across bacterial genomes. Notably, while these elements are commonly present, supernatants from certain strains containing only the Pf4 element exhibited infectivity, suggesting functional differences among these prophage-like elements (7). Additionally, studies in *P. aeruginosa* strains PAO1 and PA14 have demonstrated that prophage-like elements frequently harbor accessory genes, known as “morons,” which are not essential for the phage life cycle but can enhance bacterial virulence (18). Comparative analyses of individual phage morons have revealed their ability to modulate key bacterial phenotypes, including the suppression of twitching and swimming motilities, as well as the inhibition of virulence factor production, such as rhamnolipids and elastase (18, 19). These studies underscore the complex role of prophages in *P. aeruginosa*-type strains PAO1 and PA14, highlighting the contribution of accessory genes to bacterial virulence and prophage diversity. However, the distribution and functional significance of prophages in clinical isolates remain less well characterized, and it remains to be determined whether findings from these strains will align with previous observations.

In this study, we conducted a comprehensive analysis of the multidrug-resistant (MDR) *P. aeruginosa* clinical isolate ZS-PA-05, obtained in 2020 from the sputum of a patient with a lung infection at Zhongshan Hospital, Fudan University, Shanghai, China (20). Notably, this isolate exhibited spontaneous autolysis, suggesting the presence of a diverse and dynamic repertoire of prophages. By integrating PHASTER, VirSorter2, and CheckV analyses with targeted sequencing of prophages induced by spontaneous prophage induction (SPI), we systematically characterized the diversity and abundance of specific prophage elements. Prophages were mapped within the bacterial genome and functionally interrogated using deletion mutants to assess their impact on host physiology and underlying mechanisms. In line with previous studies, we found that prophages broadly enhance bacterial fitness traits, including growth, motility, susceptibility to lytic phage infection, and antibiotic resistance. Notably, deletion of prophage Y markedly increased host virulence and intensified its competition. These findings highlight the profound role of prophage-driven diversification in shaping host adaptive potential, with broad ecological and evolutionary implications for the management of this clinically significant pathogen.

## RESULTS

### Prophages in the genome of *P. aeruginosa* ZS-PA-05

The MDR *P. aeruginosa* strain ZS-PA-05 consistently exhibited autolysis within bacterial lawns following overnight incubation, likely driven by active prophage excision (Fig. S1). This phenomenon parallels the activation of the Pf4 prophage, where mutation of *pqsL* leads to the accumulation of 2-heptyl-4-hydroxyquinoline (HHQ), subsequently triggering Pf4 prophage induction and autolysis (21). Similarly, a comparable mechanism has been reported in quorum sensing studies of the *P. aeruginosa* PAO1 strain, where autolysis was restored by the addition of synthetic *Pseudomonas* quinolone signal (PQS) and inhibited by mutations in genes required for PQS biosynthesis (22). This observation suggests the potential for spontaneous prophage induction (SPI) events in *P. aeruginosa* strain ZS-PA-05, a process where a small subset of cells triggers prophage induction, leading to the release of mature phages. Comprehensive analysis using PHASTER, VirSorter2, and CheckV identified 12, 4, and 3 prophage-like elements in *P. aeruginosa* ZS-PA-05, respectively (Tables S1 to S3) (23–25). PHASTER predictions encompassed all prophages detected by VirSorter2 and CheckV, despite differences in predicted fragment sizes; VirSorter2 and CheckV generally identified larger regions. We selected PHASTER predictions as the reference for subsequent phenotypic analyses. Based on PHASTER annotation, five prophages were classified as intact (designated WX-1, WX-2, WX-3, WX-4, and WX-5), three as incomplete, and four as questionable (Table S1).

We next screened a collection of 32 clinical *P. aeruginosa* isolates and their corresponding mutants using cell-free supernatant (CFS) from strain ZS-PA-05 (20, 26). Notably, CFS of strain ZS-PA-05, along with that of two other strains (ZS-PA-13 and ZS-PA-14), was capable of forming plaques on *P. aeruginosa* ZS-PA-35 (Fig. S2). Following plaque formation, we harvested the plaques, extracted phage DNA, and performed high-throughput sequencing. Contrary to computational predictions, sequencing of the CFS plaques revealed three active phages in *P. aeruginosa* ZS-PA-05, designated Y, Pf1, and TY. Notably, Pf1 and TY corresponded to regions predicted by PHASTER as questionable and incomplete, yet were not classified as intact prophages (Table S1). These findings suggest that the PHASTER predictions may underestimate the completeness of Pf1 and TY and that prophage Y may represent a novel phage element not detected by the prediction algorithm. Based on the combined results of *in silico* predictions and sequencing of the CFS lysate, we selected five intact prophages identified by PHASTER, along with the three additional active phages, for further characterization in strain ZS-PA-05.

### Characterization of prophages harbored in strain ZS-PA-05

**Fig 1:**
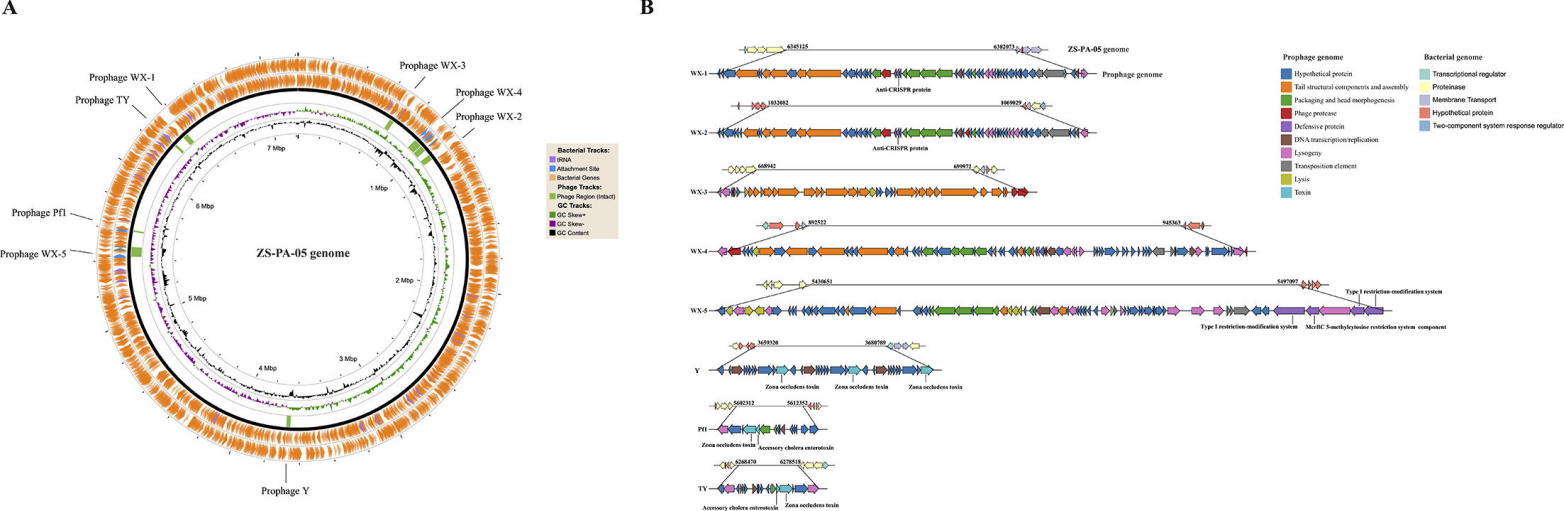
Prophage (WX-1, WX-2, WX-3, WX-4, WX-5, Y, Pf1, and TY) distribution and organization in the *P. aeruginosa* ZS-PA-05 genome. (**A**) Genome map of *P. aeruginosa* ZS-PA-05, highlighting the prophages deleted in this study. (**B**) Detailed positional distribution and annotation of the eight prophages within the chromosome of *P. aeruginosa* ZS-PA-05. Putative genes are color-coded based on the predicted functions of their products. CRISPR, clustered regularly interspaced short palindromic repeats.

All eight prophage fragments ranged in size from 10.3 to 67 kb, with guanine–cytosine (GC) contents ranging from 57.01% to 64.13%, and included 15–77 open reading frames (ORFs) (Table S4). Their precise locations within the bacterial chromosome of strain ZS-PA-05 and genome organization are highlighted in Fig. 1A and B. Notably, the genomes of all three prophages (Y, Pf1, and TY) contained zonula occludens toxin proteins, which are linked to cholera toxin production. Next, the genetic similarity among the eight prophages was analyzed using Easyfig (Fig. S3). Notably, WX-1 and WX-2 show a high degree of sequence similarity. Additionally, varying levels of similarity were observed among the other two groups (WX-3, WX-4, WX-5, and Pf1, Y, and TY); however, these similarities were relatively low.

**Fig 2:**
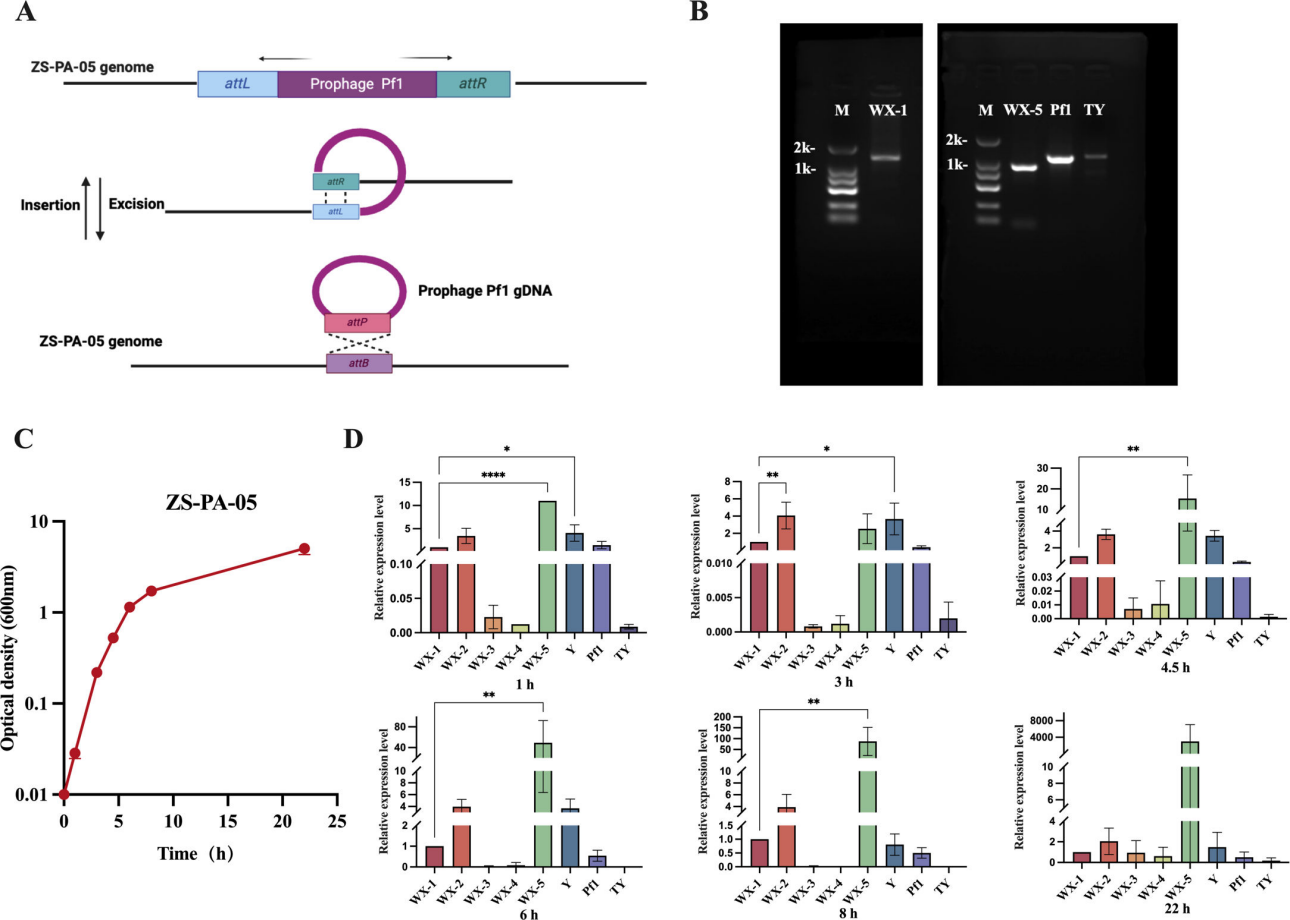
Characterization of prophage excision and integration sites in *P. aeruginosa* ZS-PA-05. (**A**) Schematic illustration of the prophage Pf1 attachment site (used as an example) in *P. aeruginosa* ZS-PA-05. (**B**) PCR detection of *attP* sites in prophages WX-1, WX-5, Pf1, and TY genomes, and *attB* sites in the host bacterial chromosome. Lane M (DNA marker, 1–2 kb), WX-1 (amplified with primers TY58 and TY59; 1,460 bp), WX-5 (amplified with primers Pa342 and Pa343, 941 bp), Pf1 (amplified with primers TY23 and TY24; 1,186 bp), and TY (amplified with primers TY27 and TY28; 1,209 bp). (**C**) CFSs were collected at various time points during the culture of *P. aeruginosa* ZS-PA-05. (**D**) Prophage induction was quantified by quantitative PCR. The relative phage numbers were normalized to the copy number of WX-1. Error bars represent standard deviations (*n* = 3). Statistical significance is indicated as follows: *P < 0.05, **P < 0.01, ****P < 0.0001.

To identify the integration sites of the eight prophages in *P. aeruginosa* ZS-PA-05, we initially selected the most likely candidates based on PHASTER predictions. These candidate sites were subsequently confirmed through PCR amplification and validated by Sanger sequencing. As shown in Fig. 2A and B, we confirmed that only prophages WX-1, WX-5, Pf1, and TY were capable of integrating into the bacterial genome through recombination at the predicted *attL* and *attR* sites (Table S5). However, despite PHASTER predicting attachment sites for WX-2, WX-3, and WX-4, we were unable to obtain bands from our PCR, suggesting that these phages may be cryptic or have excised at extremely low SPI events. For phage Y, the highly repetitive nature of its genomic sequence (Fig. S4) prevented the precise determination of its integration site by PCR. Therefore, we further aligned its sequenced genome with the bacterial chromosome to predict its attachment sites. To further investigate the relative copy numbers of the eight prophages in *P. aeruginosa* ZS-PA-05, bacterial cultures were collected at two different growth stages. The relative levels of SPI events among these phages in the supernatant at each stage were quantified by normalizing to the WX-1 phage. The results showed that prophage WX-5 exhibited the highest induction levels at 1.0, 4.5, 6.0, and 8.0 h, followed by WX-2 at 3 h and Y at 1 and 3 h. Notably, prophages WX-3, WX-4, and TY displayed relatively low induction levels (Fig. 2D).

### Deletion of prophages affects bacterial growth

**Fig 3:**
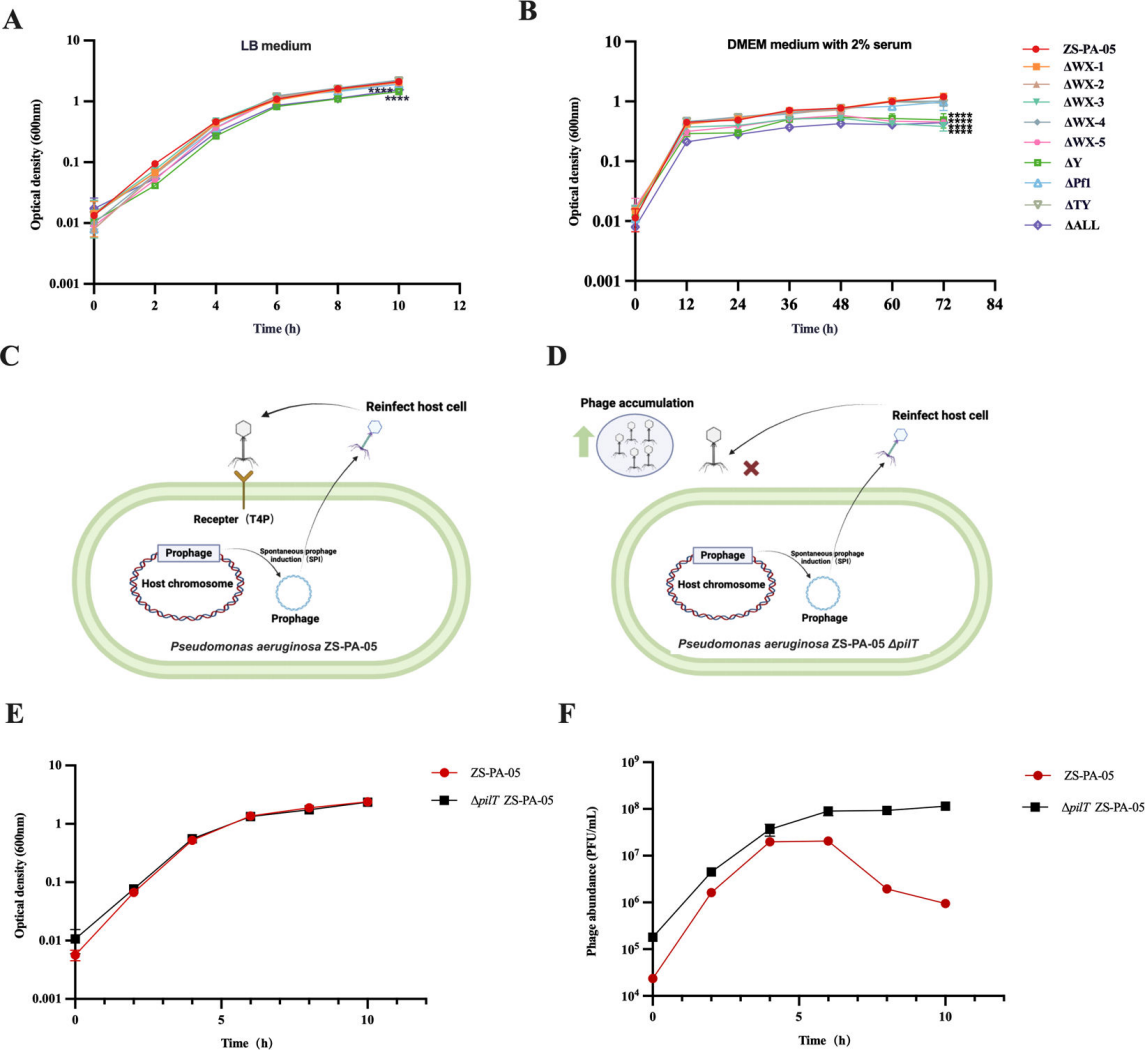
Impact of prophage deletion on bacterial growth in Luria-Bertani (LB) medium and DMEM and assessment of superinfection immunity. Growth comparison between wild-type ZS-PA-05 and its prophage knockout strains in LB medium (**A**) over a 10 h period and cell culture medium (**B**) over a 72 h period, with optical density measured at specific intervals. Schematic representation of prophage excision and superinfection dynamics in the wild-type strain ZS-PA-05 (**C**) and the receptor mutant Δ*pilT* ZS-PA-05 (**D**), respectively. (**E**) Growth curve analysis of wild-type ZS-PA-05 and Δ*pilT* ZS-PA-05 in LB medium over 10 h, with optical density at 600 nm (OD_600_) values recorded every 2 h. (**F**) Corresponding phage concentration measured in bacterial supernatant, using *P. aeruginosa* strain ZS-PA-35 as the host for phage release assessment. Error bars represent standard deviations (*n* = 3). The final point optical density was analyzed using one-way analysis of variance. Statistical significance is indicated as follows: ****P < 0.0001.

To explore the contribution of individual prophages to host phenotypes, we constructed a series of single-prophage deletion mutants: ΔWX-1, ΔWX-2, ΔWX-3, ΔWX-4, ΔWX-5, ΔY, ΔPf1, and ΔTY in *P. aeruginosa* ZS-PA-05. In parallel, we generated a mutant lacking all eight prophages (designated ΔALL) to assess their collective impact on bacterial physiology. As shown in Fig. 3A, both mutants (∆Y and ∆ALL) reached final optical densities that were only approximately half of those observed for the wild-type strain ZS-PA-05 in Luria-Bertani (LB) medium. In addition, both strains exhibited reduced average exponential growth rates in the first 4 h, with ∆Y showing approximately 0.807/h and ΔTY around 0.720/h. In contrast, deletion of other prophage regions had no discernible effect on exponential growth and, in some cases, such as ΔWX-2 (0.9945/h), ΔWX-4 (0.9368/h), and ΔWX-5 (0.9275/h), even led to significantly increased growth rates compared to the wild-type strain (0.8865/h). Also, we cultured these bacteria in Dulbecco’s modified Eagle medium (DMEM) supplemented with 2% serum to simulate *in vivo* conditions. Our results (Fig. 3B) show that the final stationary-phase optical density of all prophage knockout strains, except ∆WX-1, ∆WX-2, ∆Pf1, and ∆TY, was significantly lower than that of the wild-type strain after 72 h of incubation. The final optical densities of the four knockout strains reached only approximately half of that observed for the wild-type strain ZS-PA-05. These findings suggest that specific prophages play a critical role in promoting bacterial proliferation within the cell-culture medium (Fig. 3B). It is important to note that the phenotypic effects observed in this study cannot be solely attributed to genes encoded by the prophages. Instead, prophage excision or deletion may influence the expression of neighboring genes, thereby modulating bacterial phenotypes. Indeed, as illustrated in Fig. S5, deletion of WX-2 and WX-3 resulted in altered expression of downstream genes, whereas deletion of WX-5 affected downstream gene expression only in the ΔALL deletion mutant. In contrast, deletion of Pf1 and TY influenced upstream gene expression in the ΔALL and ΔTY mutants, respectively.

### Type IV pilus-mediated superinfection affects the accumulation of prophages

Following the CFS spot assay, no visible plaques were observed on plates inoculated with the strain Δ*pilT* ZS-PA-35, suggesting that the plaque-forming phages present in the CFS of ZS-PA-05 likely utilize type IV pilus (T4P) as their receptor (Fig. S6). However, it remains possible that other phages present in the CFS exploit alternative receptors, such as the O-antigen (27), which may result in undetectable plaques or reflect the intrinsic resistance of ZS-PA-35 to these induced phages. The concentration of induced phage particles in the culture medium depends on both the rate of phage release through prophage induction and the rate of phage loss, which is driven by how quickly the released phages adsorb to and superinfect lysogenic cells in the culture. As illustrated in Fig. 3C and D, the presence or absence of functional T4P in strain ZS-PA-05 clearly affects the accumulation of free phages in the CFS. As shown in Fig. 3E, the growth of both the wild-type ZS-PA-05 strain and the Δ*pilT* ZS-PA-05 mutant (which lacks a functional T4P receptor) in LB medium was comparable. However, distinct differences were observed in prophage dynamics (Fig. 3F). In both the wild-type and T4P mutant, once logarithmic growth was reached after 4 h, the concentration of prophages in the CFS gradually declined from approximately 10^7^ to 10^6^ PFU/mL. In contrast, the Δ*pilT* ZS-PA-05 strain exhibited a significant and continuous increase in prophage release.

### Deletion of prophages affects bacterial motilities

**Fig 4:**
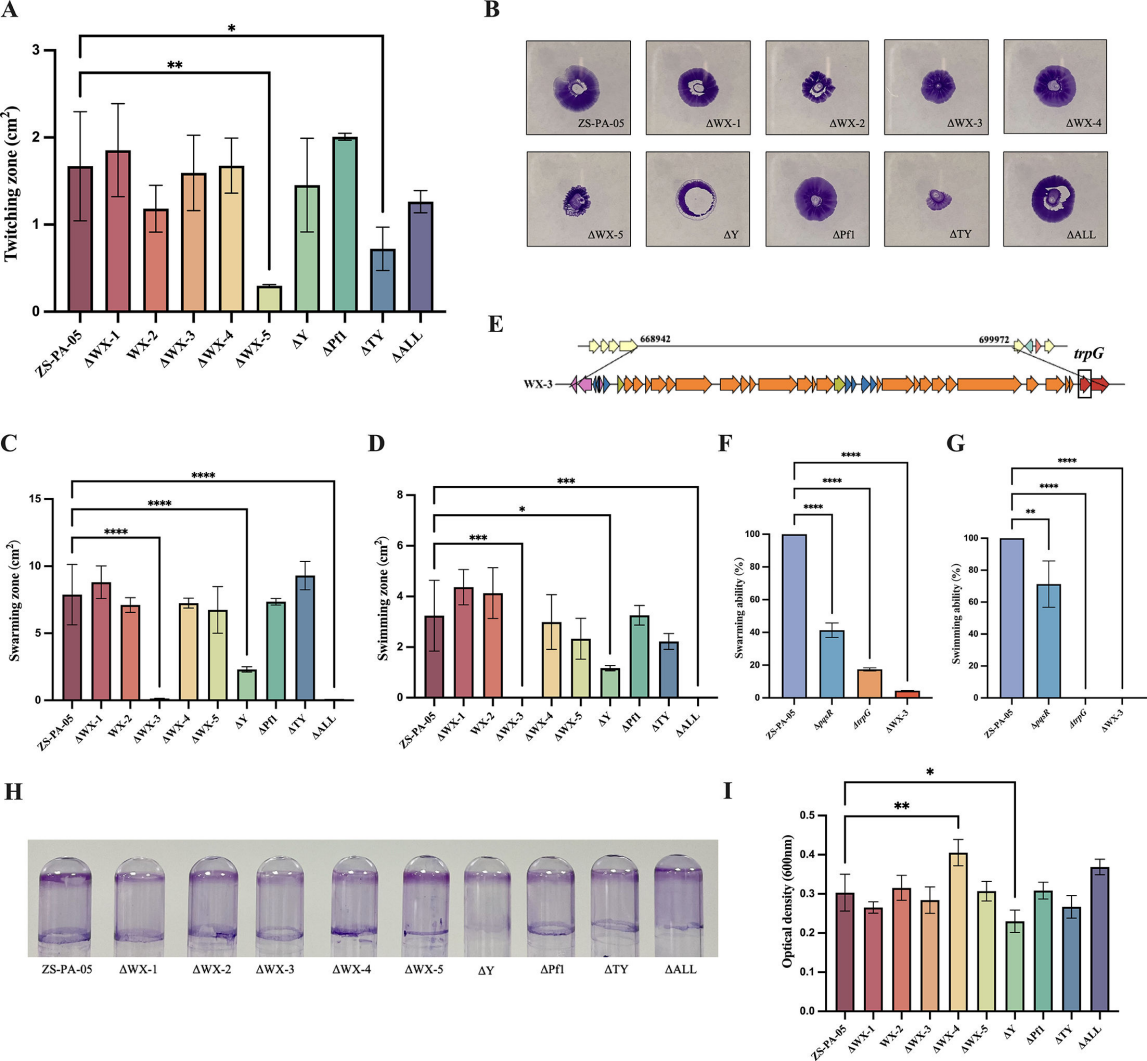
Individual prophage affects bacterial motility and biofilm formation. (**A**) Quantification of twitching motility in *P. aeruginosa* ZS-PA-05 and its prophage deletion mutants, measured by colony area (cm²) following 3 days of static incubation at 37°C. (**B**) Representative images of the twitching motility assay stained with crystal violet. (**C**) Swarming motility quantification in *P. aeruginosa* ZS-PA-05 and prophage deletion mutants, determined by colony area (cm²) after 16 h of static incubation at 37°C. (**D**) Swimming motility quantification in *P. aeruginosa* ZS-PA-05, determined by colony area (cm²) after 16 h of static incubation at 37°C. (**E**) Schematic representation of the gene sequence of prophage WX-3. The orange arrow indicates the gene encoding *trpG*. The locus of the *trpG* gene in the ZS-PA-05 genome is located between positions 698316 and 698921 bp. (**F**) Swarming motility and (**G**) swimming motility quantification in wild-type ZS-PA-05, Δ*pqsR*, and a mutant lacking *trpG*, as well as ΔWX-3. (**H**) Representative images of biofilm formation in *P. aeruginosa* ZS-PA-05 and its prophage deletion mutants visualized with crystal violet staining and quantified via optical density measurement (**I**). Error bars represent standard deviation (*n* = 3). Statistical significance is indicated as follows: *P < 0.05, **P < 0.01, ***P < 0.001, ****P < 0.0001.

In addition to T4P-mediated superinfection, motilities mediated by flagella and T4P are closely associated with the virulence of *P. aeruginosa*. These motilities play a crucial role in its movement, colonization of diverse environments, surface adhesion, and biofilm formation (28). Given this, we aimed to investigate whether prophages influence bacterial motility. Our findings revealed that compared to the wild-type strain ZS-PA-05, which exhibited an average motility area of ~1.67 cm², the motility of strains with the knocked-out prophages WX-5 (~0.287 cm²) and TY (~0.723 cm²) was markedly reduced on 1.5% LB plates (Fig. 4A and B). Similarly, studies on the filamentous phage Pf4 also indicated that the absence of prophages significantly affected the motility of strain PAO1 (29). Additionally, significant differences in flagella-mediated swarming and swimming motilities were observed on soft agar plates (0.5%). Specifically, upon knockout of prophages WX-3 and Y, both swarming and swimming motilities were notably reduced (Fig. 4C and D). Similarly, the full knockout strain ΔALL exhibited a similar reduction in motility, akin to the ΔWX-3 strain, suggesting that the observed motility reduction is primarily attributable to the absence of prophage WX-3.

The predicted WX-3 sequence encodes genes annotated as anthranilate synthase (TrpG), a key enzyme in the biosynthesis of precursor molecules for 2-heptyl-3-hydroxy-4(1H)-quinolone (Fig. 4E) (30). Disruptions in this pathway have been linked to impaired swarming motility in *P. aeruginosa* (31). To assess its functional impact, we generated a ∆*trpG* deletion mutant, as well as a deletion mutant for the regulatory factor gene *pqsR*. In line with previous findings, comparative analyses revealed a significant reduction in both swarming and swimming motility in the knockout mutants (Δ*pqsR*, Δ*trpG*, and ΔWX-3) relative to the wild type (Fig. 4F and G). These results suggest that the impaired motility observed in strain ΔWX-3 is likely due to the loss of TrpG, which perturbs tryptophan metabolism and PQS regulation, thereby affecting bacterial movement on agar.

### Deletion of prophages affects bacterial biofilm formation

Prophage contributions to *P. aeruginosa* ZS-PA-05 biofilm formation were assessed by quantifying biomass using crystal violet staining (Fig. 4H). The results revealed that the absence of prophage Y led to a significant reduction in biofilm formation compared to the wild-type strain (Fig. 4I). Interestingly, we observed that the deletion of prophage WX-4 resulted in approximately a 34% increase in biofilm formation (Fig. 4I), while the deletion of the remaining prophages had no discernible effect on biofilm formation. Taken together, these results indicate that prophages play a role in biofilm development, with varying degrees of influence among the tested prophages.

### The effect of prophages on phage susceptibilities in *P**.** aeruginosa*

**Fig 5:**
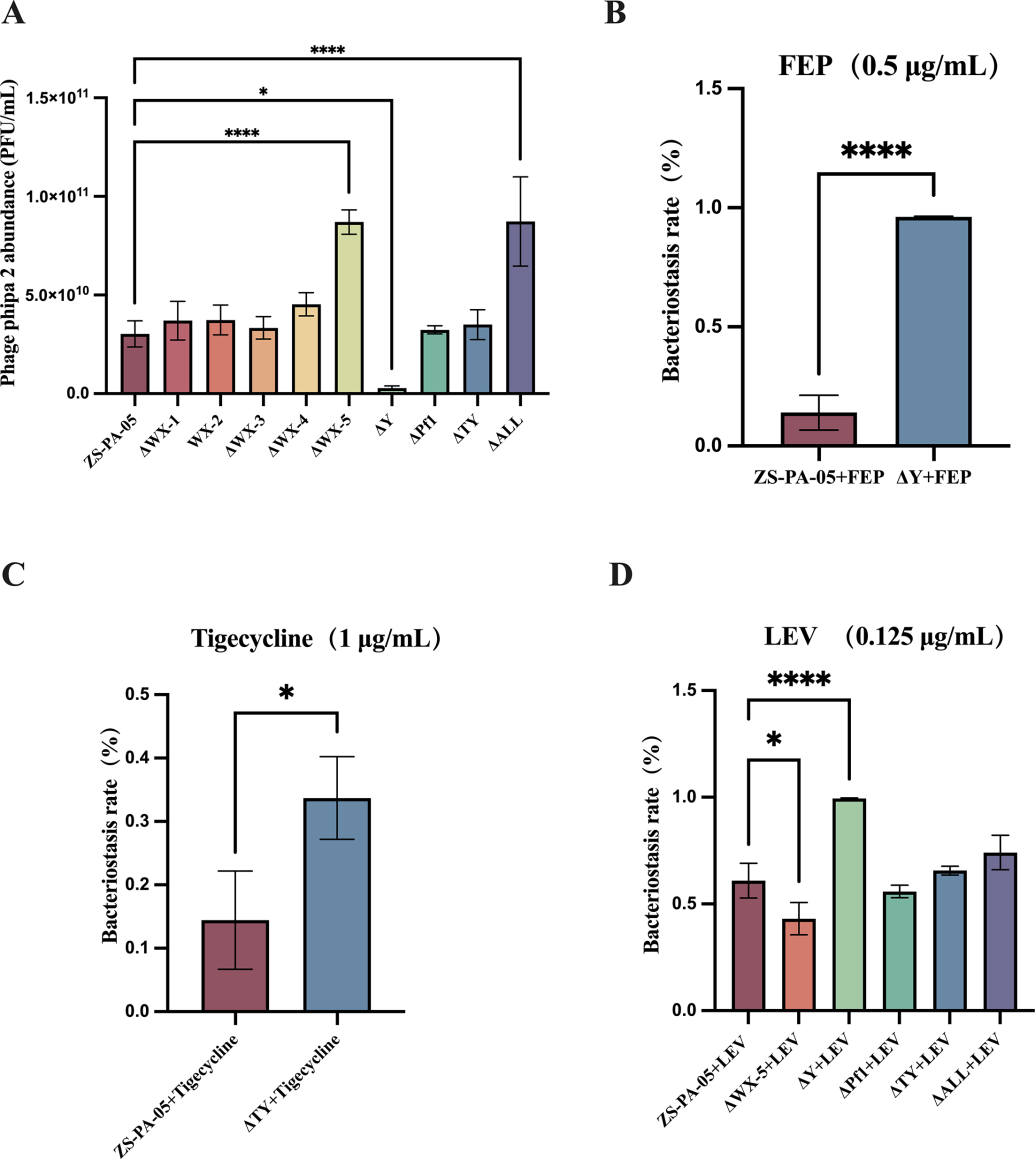
Deletion of individual prophages alters phage phipa2 proliferation and modulates antibiotic susceptibility. (**A**) Wild-type *P. aeruginosa* ZS-PA-05 and its prophage deletion mutant were cultured to logarithmic phase, followed by infection with lytic phage phipa2 and incubation at 37°C for 10 h. The impact of prophage deletion on phipa2 replication was evaluated by quantifying phage progeny production. (**B–D**) Antibiotic susceptibility assays were performed by adding a specific concentration of cefepime, levofloxacin, or tigecycline to LB medium, with the initial bacterial absorbance set at 0.001. Cultures were incubated at 37°C for 12 h, and growth of wild-type ZS-PA-05 and the prophage deletion strain was compared. Statistical significance: *P < 0.05, ****P < 0.0001. Error bars represent standard deviations (*n* = 3).

Existing literature has established a close association between mobile genetic elements and phage defense mechanisms (32). Additionally, it has been observed that the expression of certain prophages can interfere with the ability of host bacteria to be reinfected by external phages (33). Likewise, we identified genes related to the anticlustered regularly interspaced short palindromic repeats system and the restriction–modification system in the genomes of prophages WX-1, WX-2, and WX-5 (Fig. 1B). This finding suggests that prophages may act as reservoirs for bacterial immune systems. To investigate the impact of prophages on the inhibition of lytic bacteriophages, we initially selected eight lytic phages from our laboratory and examined their lytic activity against the wild-type strain ZS-PA-05 and corresponding prophage mutants (20). Our findings revealed that among the eight phages tested, only those targeting T4P exhibited lytic activity (Fig. S7). Subsequently, we focused on phipa2, specifically assessing host sensitivity and progeny release. Phage titers were measured after introducing phipa2 to cultures during the logarithmic growth phase and co-incubating for 10 h. The results indicated that the knockout of prophage Y significantly reduced phipa2 progeny phage release, showing a 10-fold decrease at 10 h. In contrast, the knockout of prophage WX-5 markedly enhanced phipa2 progeny release, resulting in a threefold increase at the same time point. Additionally, the simultaneous knockout of all prophages produced an effect on phipa2 progeny phage release similar to that of the WX-5 knockout (Fig. 5A).

### The effect of prophages on antibiotic susceptibility in *P**.** aeruginosa*

To assess the impact of prophages on bacterial antibiotic susceptibility, we evaluated the sensitivity of *P. aeruginosa* ZS-PA-05 and its corresponding prophage deletion mutants to clinically relevant antibiotics. Minimum inhibitory concentrations (MICs) were determined using the VITEK 2 system. Our results showed that the deletion of certain prophages significantly altered the sensitivity of the wild-type strain ZS-PA-05 to three antibiotics: cefepime, tigecycline, and levofloxacin (Fig. S8A and B). Based on these findings, these three antibiotics were selected for further investigation. Specific concentrations of cefepime (0.5 µg/mL), tigecycline (1 µg/mL), and levofloxacin (0.125 µg/mL) were applied, and bacterial growth was monitored. The results indicated that, compared to the wild-type strain, the growth inhibition by cefepime and levofloxacin was significantly enhanced in the ΔY prophage knockout strain, while levofloxacin’s inhibition was significantly reduced in the ΔWX-5 knockout strain. Moreover, tigecycline showed a notably increased inhibition rate in the ΔTY knockout strain (Fig. 5B through D). Although no antibiotic resistance genes were predicted in the genomes of the eight prophages studied, our findings demonstrate that the presence of prophages significantly influences the antibacterial activity of antibiotics against *P. aeruginosa* ZS-PA-05.

### Deletion of prophage Y improves its interspecies competition ability

**Fig 6:**
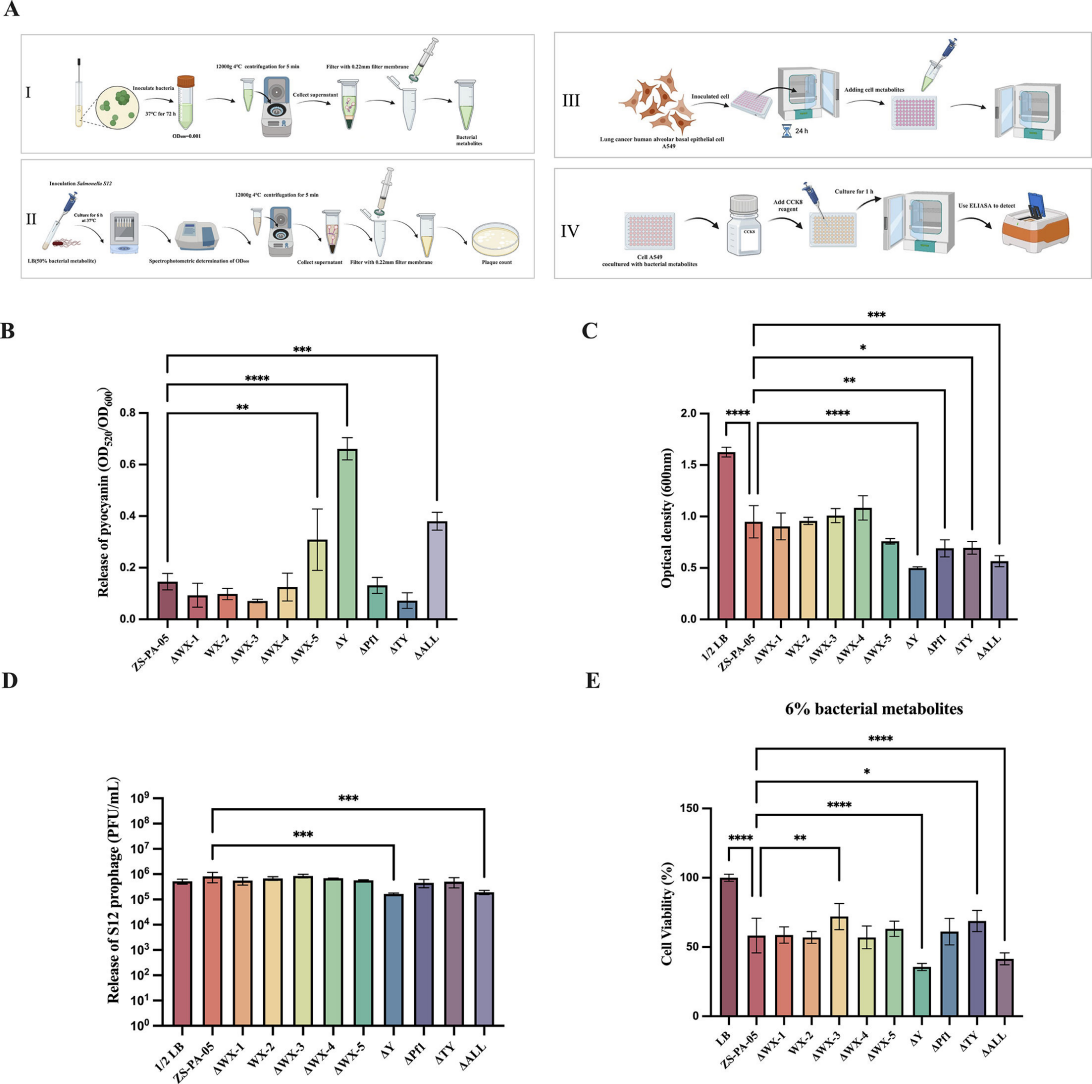
Impact of prophages on pyocyanin release, bacterial competitiveness, and virulence. (**A**) Schematic overview of the experimental workflow: (I) quantification of pyocyanin production, (II) addition of bacterial metabolites to *Salmonella* spp*.* S12 to assess growth and prophage induction; and (III and IV) culture of A549 cells to evaluate host cell survival. (**B**) Pyocyanin levels were quantified from CFS of *P. aeruginosa* ZS-PA-05, and its prophage deletion mutants were cultured at 37°C for 3 days, initiated at an optical density (OD_600_) of 0.001. (**C**) The interspecies effect of CFS from *P. aeruginosa* ZS-PA-05 and its prophage knockout strains was assessed by adding 50% bacterial metabolites to *Salmonella* spp*.* S12, followed by incubation at 37°C for 6 h. Bacterial growth (**C**) and *Salmonella* prophage release (**D**) in S12 were measured using *Salmonella* spp. S3 as the host, with the solution containing 50% 1× phosphate-buffered saline. (**E**) To assess the impact on human cells, bacterial CFS was added to A549 cell cultures at a final concentration of 6% and incubated at 37°C for 10 h. Cell survival was then measured using the CCK-8 assay. Statistical significance: *P < 0.05, **P < 0.01, ***P < 0.001, ****P < 0.0001. Error bars represent standard deviations (*n* = 3).

*P. aeruginosa* enhances toxicity production through the production of redox-active phenazines, such as pyocyanin (34). Previous studies have also shown that Pf filamentous phages increase the virulence of *P. aeruginosa* by regulating pyocyanin secretion (35). To assess the impact of prophages on bacterial virulence, we first examined the effect of prophage knockout on pyocyanin production (Fig. 6A). Our results indicate that pyocyanin production was significantly increased in *P. aeruginosa* ZS-PA-05 following the knockout of prophages WX-5 and Y. A similar trend was observed in the strain ΔALL, though pyocyanin release in the full knockout was surprisingly lower than that in ΔY (Fig. 6B).

In line with previous findings, phenazine pyocyanin has been identified as a potent inducer of *Staphylococcus aureus* prophages, showing more selective induction compared to mitomycin C (36). We sought to determine whether these metabolites could trigger prophage release in neighboring strains. We collected bacterial metabolites from all 10 strains and inoculated lysogenic *Salmonella* S12 into LB medium supplemented with 50% of these metabolites. Both the growth of *Salmonella* spp*.* S12 and prophage release were monitored. As shown in Fig. 6C and D, supplementation with 50% bacterial metabolites from *P. aeruginosa* ZS-PA-05 or its prophage deletion mutants significantly affected *Salmonella*’s growth and prophage induction compared to the control group supplemented with LB medium instead of CFS. The bacterial metabolites from the ΔY and ΔALL prophage knockout strains had a greater impact on the growth of *Salmonella* spp. S12 and prophage release compared to the wild-type strain ZS-PA-05. However, contrary to expectations, pyocyanin production in ZS-PA-05 did not enhance prophage release in *Salmonella* spp*.* S12; instead, it decreased. Perhaps, this suggests that pyocyanin may have a selective induction effect on prophages.

### Effect of prophages on bacterial virulence

Hospital-acquired pneumonia remains a significant cause of mortality among critically ill patients suffering from nosocomial infections (37). In light of its clinical importance, we selected the A549 human alveolar basal epithelial cell line, commonly used as a lung cancer model, to investigate the effects of prophage knockout on the virulence of *P. aeruginosa* ZS-PA-05. We collected bacterial metabolites from *P. aeruginosa* ZS-PA-05 and its prophage knockout strains and co-cultured them with A549 cells for 10 h using 6% metabolite concentration gradients. The survival of A549 cells was then compared between the wild-type ZS-PA-05 and the prophage mutants (Fig. 6E). After 10 h of co-culture, the survival rate of A549 cells exposed to wild-type bacterial metabolites was 58.35%, significantly lower than the LB blank control group, indicating strong cytotoxicity of *P. aeruginosa* ZS-PA-05 toward A549 cells. Furthermore, we observed that the viability of cells cultured with metabolites from ΔY and ΔALL was significantly lower than those cultured with wild-type strain metabolites. Conversely, cell viability was higher when cultured with metabolites from ΔWX-3 and ΔTY knockout strains compared to the wild-type strain. These findings suggest that different prophages have varying effects on the virulence of ZS-PA-05, either enhancing or reducing its cytotoxicity *in vitro*. One possibility is that the virulence of *P. aeruginosa* ZS-PA-05 is strongly linked to pyocyanin production, with elevated pyocyanin levels correlating with increased virulence. However, given the complexity of the pathogenic network and the presence of multiple virulence factors, we cannot exclude the likelihood that other factors also contribute to the modulation of virulence in ZS-PA-05.

## DISCUSSION

**Fig 7:**
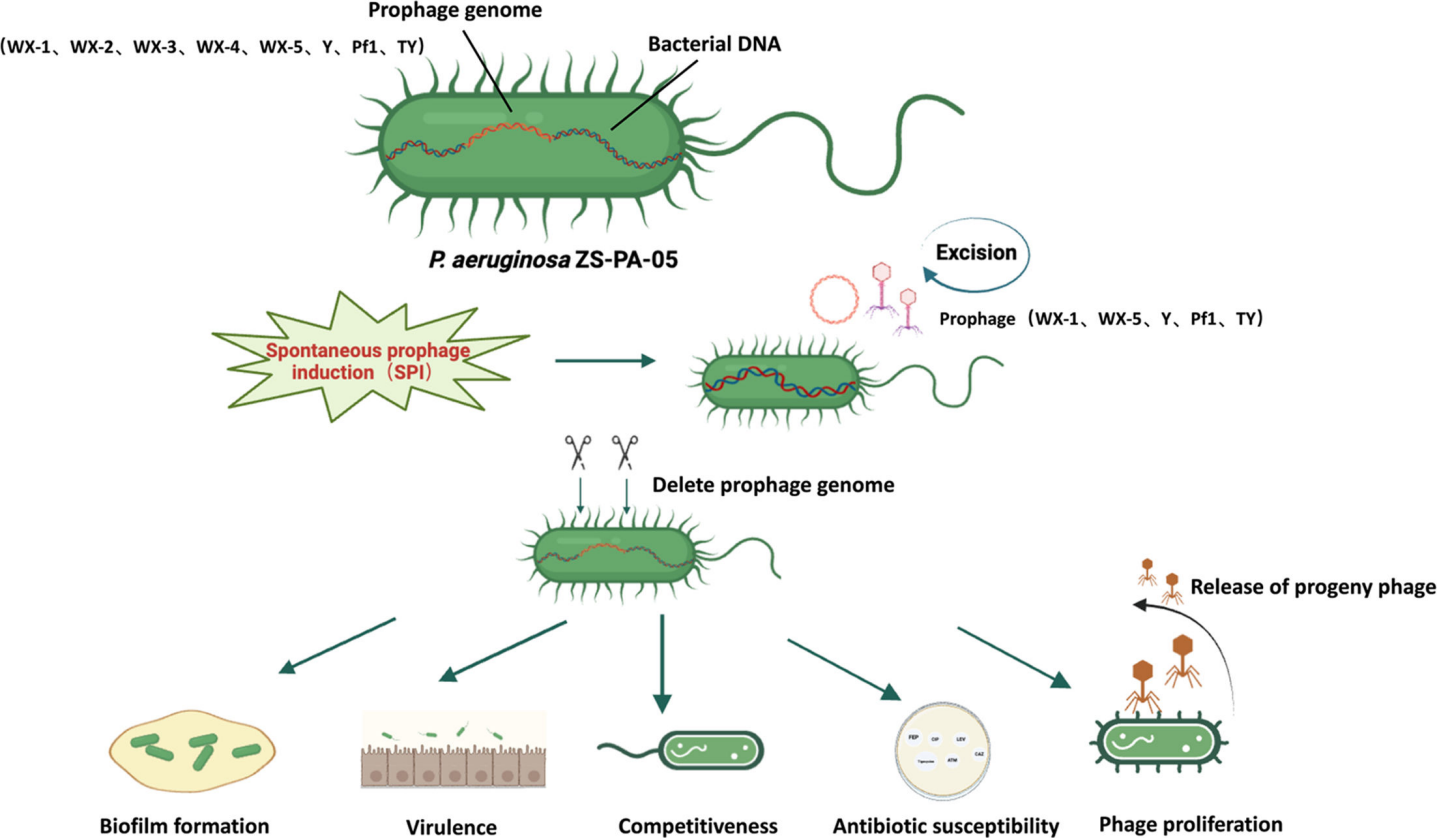
Schematic illustration of the impact of individual prophages on *P. aeruginosa* ZS-PA-05 physiology. Prophage deletion influences multiple bacterial traits, including growth, motility, biofilm formation, phage susceptibility, antibiotic sensitivity, intraspecies competition, and virulence.

Prophages, as accessory elements of bacterial genomes, play a pivotal role in shaping bacterial traits, driving evolution and diversification. Studies have shown that active prophages can influence not only individual cells but also entire bacterial communities, affecting key aspects of physiology, metabolism, and evolutionary dynamics (17). In this study, we performed a comprehensive analysis of eight prophages in *P. aeruginosa* ZS-PA-05, systematically characterizing their composition, dynamics, and distribution. This work provides new insights into phage biology and deepens our understanding of the complex interactions between prophages and their bacterial host, as illustrated in Fig. 7.

To elucidate the functional roles of prophages and their impact on host adaptability, we generated prophage deletion mutants and compared them to the wild-type *P. aeruginosa* ZS-PA-05 strain. Adjacent gene expression analysis revealed that the phenotypic effects associated with certain prophages extend beyond the functions of their encoded genes. Rather, prophage excision or deletion can influence the expression of adjacent bacterial genes, potentially shaping key phenotypic traits. However, the extent to which these transcriptional changes directly contribute to phenotypic variation remains to be determined. Supporting this observation, a study on the closely related *P. aeruginosa* P9W strain demonstrated that RecT promotes the excision of prophage PP9W by inhibiting the CI-like repressor pg40, leading to the derepression of transcription factors and essential genes involved in cellular processes (38). This regulatory cascade enhances bacterial growth, biofilm formation, and virulence. Similarly, in *Listeria monocytogenes*, prophage excision has been shown to activate the Com system by restoring the functional *comK* gene, serving as a genetic switch to modulate virulence during infection (39). Taken together, our findings underscore the critical role of prophages in shaping *P. aeruginosa* pathogenicity and adaptive growth but also highlight their broader significance in influencing bacterial gene expression.

Bacterial cell structure integrity and protective extracellular polysaccharide capsules play a crucial role in biofilm formation, which is essential for persistent bacterial infections. The extracellular matrix, secreted during prophage-induced lysis, is a complex structure composed of extracellular polysaccharides, further contributing to the stability and persistence of surface-associated microbial communities (40). This matrix forms an interactive scaffold that supports the biofilm structure and facilitates cell communication. Research has shown that prophages can significantly impact biofilm development, biofilm dispersion, and colony variation (41). For example, *P. aeruginosa* PAO1 biofilms actively produce Pf phages, which self-assemble into a highly ordered liquid crystalline matrix. This structured matrix reinforces biofilm integrity, promoting bacterial adhesion and resilience while enhancing tolerance to desiccation and antibiotic stress (42). Similarly, prophage Pf5 has been shown to enhance biofilm formation through the substrate-binding protein DppA1, which inhibits Pf5 lysis (41). Our study unveils divergent effects of prophages on biofilm formation. Deletion of prophage WX-4 enhanced biofilm formation, whereas loss of prophage Y reduced it, underscoring the opposing roles of distinct prophages in modulating biofilm dynamics. This variability reflects the complex and context-dependent nature of prophage-mediated regulation of biofilm development.

Interestingly, both prophages WX-3 and WX-4 may have lost the ability to excise spontaneously from the host genome, remaining in a permanent state of lysogeny. The genes within WX-3 have been repurposed to benefit the host rather than the phage itself (17), enhancing bacterial resilience under environmental stress. This adaptation is evidenced by improved growth in amino acid-minimal media and enhanced metabolism and utilization of tryptophan (43). Consequently, increased motility facilitates bacterial dispersal from biofilms, thereby reducing biofilm formation. Similarly, deletions of prophages WX-5 and Y inhibit twitching motility, likely through indirect effects on T4P expression. Together, our findings highlight the intricate interplay between receptor-mediated motility and prophage dynamics. Unlike our study, previous studies have mechanistically examined prophage-mediated defense against phage infections via T4P. Specifically, the Pf superinfection exclusion protein PA0721 confers resistance to Pf infection by suppressing twitching motility through its interaction with the T4P protein PilC (29). However, paradoxically, during interactions between the lytic phage phipa2 and prophage deletion mutants, we observed that reduced twitching motility in ΔWX-5 was associated with an increase in phage progeny production, a trend not observed in the ΔY mutant. One possible explanation is that phage strains with a higher adsorption rate may exhibit a shorter optimal lysis time (44). Early bacterial lysis is a well-documented phenomenon, known as lysis from without, under high multiplicity of infection, where cell contents are released due to the rupture and degradation of the cell wall, and adsorbed phages are not recovered following lysis (45).

From a therapeutic perspective, prophage-mediated defense mechanisms and phage receptor regulation could hinder the efficacy of lytic phage therapy in combating MDR infections (46). Beyond stand-alone phage therapy, lytic phage-antibiotic combinations offer promising strategies for treating MDR, extensively drug-resistant, and difficult-to-treat *P. aeruginosa* infections (47). However, several challenges remain in the clinical application of such therapies. Prophages function as regulatory switches, modulating bacterial genes through genomic excision and impacting bacterial adaptability, including susceptibility to antibiotics and phages (48). Here, we find that prophage deletions led to altered bacterial sensitivity to lytic phages. Similarly, P4-like prophages in *Escherichia coli* have been shown to contain genetic variation hotspots, including antiphage systems (49). Additionally, we observed changes in antibiotic susceptibility following prophage deletions, though the precise mechanisms remain unclear. While antibiotic resistance is often disseminated via horizontal gene transfer on mobile genetic elements, including prophages, few integrative phages are reported to carry antibiotic resistance genes (50). However, none of the eight prophages identified in the genome of *P. aeruginosa* ZS-PA-05 encoded antibiotic resistance genes. Further research is needed to unravel the mechanisms by which prophages influence bacterial antibiotic resistance. The carrying of prophages can also affect bacterial virulence in various ways, including the secretion of phage-encoded toxins, modulation of bacterial membranes, mediation of infectivity, and control of bacterial cell regulation (51). Here, when we cultured cells with the sterile bacterial metabolic products, we observed varying effects on cell survival, suggesting that certain prophages significantly impact bacterial virulence and competitiveness.

To advance scientific understanding and inform therapeutic strategies, it is crucial to elucidate the regulatory roles of these eight prophages on the genotype and phenotype of the clinically MDR pathogen *P. aeruginosa* ZS-PA-05. Investigating the co-evolution and interactions between these prophages and their bacterial hosts will enhance our knowledge of host–phage dynamics and their potential applications in clinical treatment. Our study demonstrated that different prophages exert varying degrees of influence on the biological phenotypes of *P. aeruginosa* ZS-PA-05. This investigation focused exclusively on the phenotypic changes mediated by the eight prophages identified to date. It is possible that other prophages remain unidentified. Future studies are needed to investigate how the eight co-existing prophages in *P. aeruginosa* ZS-PA-05 compete for cellular resources in response to common triggers. Additionally, exploring their distribution in clinical samples could provide valuable insights into their broader impact on *P. aeruginosa* population dynamics and community structure. Nevertheless, our findings enhance the understanding of the functional roles of multiple prophages in clinically isolated *P. aeruginosa*, revealing that distinct prophages within the same bacterial strain mediate different biological responses.

## MATERIALS AND METHODS

### Bacterial strains, media, and growth conditions

The bacterial strains and plasmids utilized in this study are detailed in Table S6. *P. aeruginosa* ZS-PA-05 and ZS-PA-35 were isolated from sputum samples collected from a patient in the infectious diseases department of a hospital in Shanghai, China. *Salmonella* spp*.* strains S12 and S3 were obtained from our laboratory collection. Unless otherwise specified, all strains were cultured in LB liquid medium (10 g/L NaCl, 10 g/L tryptone, and 5 g/L yeast extract) or on LB agar plates (15 g/L agar) at 37°C. Antibiotics were used at the following concentrations: triclosan at 100 mg/mL for *P. aeruginosa* and gentamicin at 15 and 30 mg/mL for *E. coli* and *P. aeruginosa*, respectively.

### Isolation and sequencing of bacterial and phage DNA

Genomic DNAs from bacterial strains were extracted using the Wizard Genomic DNA Purification Kit (Promega, Madison, WI, USA) following the manufacturer’s protocol. The extracted DNA was prepared for PacBio sequencing at the Chinese National Human Genome Center (Shanghai, China). ORFs in the bacterial genome were annotated using the RAST server (https://rast.nmpdr.org/). Potential prophage gene sequences were identified by submitting the bacterial genome sequence to the PHASTER (http://phaster.ca/), VirSorter2, and CheckV websites (23–25).

Phage DNA was extracted with the Qiagen DNeasy Blood and Tissue Kit (Qiagen, California, USA). Briefly, bacterial genomic DNA and RNA were removed from phage lysates by treatment with DNase I and RNase A (Thermo Scientific, California, USA) for 1.5 h at 37°C. Enzymes were inactivated with EDTA (final concentration 20 mM) and heat at 80°C for 5 min, after which the manufacturer’s protocol was followed. Sequencing was conducted at the Chinese National Human Genome Center using an Illumina platform. Briefly, a 100 ng sample of DNA was used to construct the library. The DNA was sheared into 400 bp fragments using a Covaris S2 instrument (Covaris, USA). The fragmented DNA was then used for library preparation with the NEXTflex DNA Sequencing Kit, compatible with the Biomek FXp system (Bio Scientific, USA). Approximately 1 Gbp of sequencing data were assembled using Newbler, resulting in the finalized phage genome sequence. The resultant prophage sequences were annotated for ORFs using the RAST server, and predicted protein functions were assessed through BLASTp against the non-redundant protein database (https://blast.ncbi.nlm.nih.gov/Blast.cgi). Additionally, the functions of ORFs encoded by prophage genes were analyzed using the PHASTER, VirSorter2, and CheckV. Comprehensive information on potential prophages in *P. aeruginosa* ZS-PA-05 and their encoded ORFs was compiled and categorized. These eight prophages were selected for comparison of genomic characteristics using Easyfig, with a minimum nucleotide sequence identity of 70% (52).

### Prophage attachment site analysis

Prophage integration sites in ZS-PA-05 were predicted using PHASTER-identified attachment sites and validated by BLASTn analysis (https://blast.ncbi.nlm.nih.gov/blast.cgi) of prophage terminal sequences. Conserved regions exhibiting near-perfect sequence identity (~100%, allowing minor SNP variations) were designated as candidate integration sites. Out-forward facing primers were designed to be positioned 500–600 bp upstream of the predicted attL and attR sites on the prophage genome (primer details are provided in Table S8). PCR was carried out in a 25 µL reaction containing 12.5 µL DreamTaq PCR Master Mix (2×), 0.625 µL of each primer pair (final concentration 0.2 µM; prophage WX-1: TY58/TY59, prophage WX-5: Pa342/Pa343, prophage Pf1: TY23/TY24, and prophage TY: TY27/TY28), 0.25 µL of ZS-PA-05 genomic DNA, 1.25 µL of 5% dimethyl sulfoxide, and 9.75 µL of sterile deionized water. Thermal cycling conditions were as follows: initial denaturation at 95°C for 3 min, followed by 45 cycles of denaturation at 95°C for 30 s, annealing for 2 min 30 s, extension at 72°C for 30 s, and a final extension at 72°C for 5 min. The PCR product was purified and subjected to Sanger sequencing.

### Bacterial genome manipulation

Prophage deletion mutants (ΔTY) and gene knockouts of *pqsR* and *trpG* were generated by allelic exchange in *P. aeruginosa* ZS-PA-05 following a previously established method for large-fragment deletions in *P. aeruginosa* ZS-PA-35 (20, 53). Briefly, the upstream and downstream regions flanking the prophage were amplified using the genomic DNA of strain ZS-PA-05 as the template. The resulting fragments, containing a 30 bp overlapping identical sequence, were then fused by overlap extension PCR. The resultant fusion PCR product was digested, purified, and inserted into the plasmid pEXG2, which was then introduced into competent *E. coli* SM10 cells by heat shock. Recombinant plasmids were selected on 1.5% LB agar plates supplemented with 15 µg/mL gentamicin. These plasmids were subsequently conjugated from the donor *E. coli* strain SM10 into *P. aeruginosa* ZS-PA-05. The conjugated strains were selected on 1.5% LB agar plates containing 100 µg/mL triclosan and 30 µg/mL gentamicin. Prophage deletion mutants were further isolated on agar plates containing 10% sucrose (5 g/L NaCl, 5 g/L tryptone, 1 g/L yeast extract, 15 g/L agar, and 100 g/L sucrose) and incubated at room temperature for 48 h. The presence of the desired chromosomal deletions was confirmed by PCR and Sanger sequencing. Genomic DNA from all prophage mutants was extracted using the Wizard Genomic DNA Purification Kit (Promega) according to the manufacturer’s protocol. The extracted DNA was prepared for Illumina sequencing at Sangon Biotech (Shanghai, China), and the sequence data were compared to that of the wild-type strain ZS-PA-05 to validate the deletion of the prophage gene sequences properly. For the construction of ΔWX-1, ΔWX-2, ΔWX-3, ΔWX-4, ΔWX-5, ΔY, and ΔPf1 mutants, fusion of PCR products was unsuccessful due to the high GC content. Instead, the flanking regions were synthesized (Sangon Biotech), and the protocol was followed from the splice overlap extension (SOEing) step. For the ΔALL mutant, the corresponding plasmids were conjugated sequentially into the recipient strain as described above.

### Quantification of adjacent gene expression following prophage deletion

To assess potential polar effects of prophage genome deletion on adjacent gene expression, we performed reverse transcription quantitative PCR analysis targeting genes located upstream and downstream of the deleted regions. Overnight cultures of the wild-type strain ZS-PA-05 and corresponding prophage-deletion mutants were inoculated into fresh LB medium at an initial optical density at 600 nm (OD_600_) of 0.1 and grown to mid-log phase (OD_600_ = 0.3). Total RNA was extracted using Trizol (Thermo Fisher Scientific) and reverse-transcribed to cDNA as described previously (26). Each 20 µL reverse transcription reaction comprised 500 ng RNA template, 2 µL random primers, 4 µL 5× reaction buffer, 2 µL 10 mM deoxynucleotide triphosphate mix, 1 µL RevertAid M-MuLV RT (200 U/µL), and 1 µL diethyl pyrocarbonate-treated water. The reaction was incubated at 25°C for 5 min, 42°C for 60 min, and terminated at 70°C for 5 min. Quantitative PCR was performed in 25 µL reactions containing 12.5 µL SsoAdvanced SYBR Green Supermix (Bio-Rad), 0.5 µL each of forward and reverse primers, 5 µL cDNA template, and 6.5 µL nuclease-free water. Amplification was carried out on an Applied Biosystems 7500 Real-Time PCR system under the following conditions: 95°C for 30 s, followed by 39 cycles of 95°C for 5 s and 55°C for 30 s, with melt-curve analysis from 65°C to 95°C at 0.5°C increments. Gene expression was quantified for loci flanking each prophage region in the wild type, individual deletion mutants (ΔWX-1, ΔWX-2, ΔWX-3, ΔWX-4, ΔWX-5, ΔY, ΔPf1, and ΔTY), and the complete deletion strain (ΔALL). Expression levels were normalized to the reference gene *rpoD*, and relative quantification was calculated based on cycle threshold (Ct) values (54).

### Detection of relative prophage copy numbers in bacterial CFS

To quantify the relative copy numbers of induced phages in bacterial CFS, overnight cultures were subcultured into fresh LB medium, with the initial optical density (OD) adjusted to 0.01. Bacterial cultures were sampled at six distinct time points corresponding to different growth phases. CFS was obtained by centrifugation and filtration through a 0.22 µm sterile syringe filters (polyvinylidene difluoride [PVDF]; Millipore, USA). Total phage DNA was extracted from the CFS using the Qiagen DNeasy Blood and Tissue Kit, diluted to 0.01 ng/µL, and subjected to quantitative PCR (qPCR) analysis with two concentration gradients. Each 25 µL qPCR reaction contained 12.5 µL of SYBR Green Master Mix, 0.5 µL of each forward and reverse primer, 5 µL of DNA template, and 6.5 µL of sterile water. Primer sequences were designed using the PrimerQuest Tool (Integrated DNA Technologies). The qPCR program was set as follows: an initial denaturation at 95°C for 30 s, followed by 39 cycles of denaturation at 95°C for 5 s, annealing at 55°C for 30 s, and a melt-curve analysis from 65°C to 95°C with 0.5°C increments every 5 s. The qPCR Ct values were used to determine the relative abundance of each prophage in the total phage DNA pool over time. To normalize variations in phage induction, *Salmonella* phage PSAP2 (3 × 10⁷ PFU/mL) was spiked into CFS as an internal reference, and its Ct value was used as a benchmark for calculating prophage abundance in the supernatant. Primers used in the study are listed in the Supplemental Materials (Table S8). Each experiment was performed in triplicate.

### T4P-mediated phage superinfection

Overnight cultures of ZS-PA-05 and ZS-PA-05 Δ*pilT* were diluted in LB medium to an initial OD_600_ of 0.01 and incubated at 37°C with shaking for 10 h. Samples were collected every 2 h, and the supernatants were harvested by centrifugation at 16,000 × *g* for 2 min at 4°C. Bacterial growth was monitored, and phage titers (PFU/mL) in the supernatants were quantified using ZS-PA-35 as the indicator strain. All experiments were conducted in biological triplicate.

### Bacterial exponential growth measurement

The overnight cultures of ZS-PA-05 and its prophage deletion mutants were diluted in liquid medium to an initial optical density of 0.01. The cultures were incubated at 37°C with shaking for a certain period of time, with samples collected at specific intervals. Bacterial exponential growth rates were determined using the formula $\;\mu =\frac{\ln\left(DO\right)-ln\left({DO}_{i}\right)}{t-t_{i}}$, where ${DO}_{i}$ is the OD at 0 h, DO is the OD at 4 h, and $t-t_{i}$ = 4h (55). Three independent replicates were performed for each experiment.

### Bacterial motility and biofilm formation assays

Biofilm formation was measured according to the methods reported in reference 56. Swarming and swimming motility assays were performed according to the methods reported in references 31 and 57. Overnight cultures of ZS-PA-05 and its prophage deletion mutants were adjusted to an OD_600_ of 1.0 and diluted in LB liquid medium. For swarming motility assessment, 2 µL of the bacterial suspension was inoculated onto the surface of 0.5% agar plates (containing 1 mM MgSO_4_, 8.6 mM NaCl, 1 mM CaCl_2_, 20 mM NH_4_Cl, 22 mM KH_2_PO_4_, 12 mM Na_2_SO_4_, 0.2% glucose, and 0.5% casein amino acids), prepared in advance in a 20 mL volume. For swimming motility analysis, bacterial suspensions were stab-inoculated into 0.3% agar plates composed of 10 g/L tryptone, 5 g/L NaCl, 3 g/L agar, and 20 g/L glucose. After 16 h incubation at 37°C, the motility zones were photographed and quantified using ImageJ software (https://imagej.net/ij/).

### Phage proliferation assay

Lytic phage host range and phage proliferation assays were performed on *P. aeruginosa* strain ZS-PA-05 and its derivative mutants. Overnight cultures of ZS-PA-05 and its prophage mutants were diluted in LB broth to an initial OD of 0.3. Subsequently, 10 µL of lytic phage phipa2 was added to each culture, followed by incubation at 37°C with shaking for 10 h. Phage proliferation was quantified using ZS-PA-05 as the host to exclude interference from any prophages.

### Antibiotic sensitivity test

Antibiotic susceptibility was assessed using the VITEK 2 system (8.01) for the following antibiotics: ticarcillin/clavulanic acid, piperacillin, ceftazidime, cefoperazone, cefepime, aztreonam, imipenem, meropenem, amikacin, tobramycin, ciprofloxacin, levofloxacin, tigecycline, and colistin. Antibiotics that exhibited differential inhibitory effects on the prophage deletion mutants compared to the wild-type strain were selected for further evaluation using broth microdilution susceptibility testing. The range of antibiotic concentrations was determined based on the MIC values. Briefly, overnight cultures of *P. aeruginosa* ZS-PA-05 and its prophage deletion mutants were diluted 1,000-fold in LB medium and grown at 37°C with shaking until the OD reached 1. Various concentrations of antibiotics were then added to the LB medium and mixed thoroughly. Bacterial suspensions were diluted to an OD of 0.001 and added to the antibiotic-containing LB medium. After 12 h of incubation at 37°C with shaking, bacterial growth was assessed to identify the antibiotic concentrations that resulted in significant differences in inhibition rates between the wild-type strain and the prophage deletion mutants. All experiments were conducted in triplicate.

### Pyocyanin extraction and semi-quantitative analysis

Pyocyanin was extracted according to previously described methods with slight modifications (58). Briefly, overnight cultures of *P. aeruginosa* ZS-PA-05 and its prophage deletion mutants were diluted in 6 mL of LB medium to an initial optical density (OD_600_) of 0.001 and incubated at 37°C with shaking for 72 h. Bacterial growth was monitored, and 5 mL of each culture was centrifuged at 12,000 × *g* for 3 min at 4°C. For pyocyanin extraction, 4 mL of the resulting supernatant was mixed with an equal volume of chloroform and vortexed for 10 s. After allowing the mixture to stand for 20 min, the lower organic phase (3 mL) was collected and combined with 1.5 mL of 0.2 M HCl, followed by another 10 s of vortexing. After an additional 20 min, the upper pink aqueous phase (1 mL) was collected, and its absorbance at 520 nm (OD520) was measured. Pyocyanin production was quantified as the OD_520_/OD_600_ ratio.

### Competitive growth assay

For the competitiveness assay, CFS was obtained by filtering the bacterial culture through 0.22 µm sterile syringe filters (PVDF, Millipore). Overnight cultures of *Salmonella* strain S12 were diluted in LB medium supplemented with 50% bacterial metabolites (1 mL of LB medium with 50% 1× PBS as a blank control) to an initial OD_600_ of 0.1. The cultures were incubated at 37°C with shaking for 6 h, after which bacterial optical density was monitored. Prophage release in *Salmonella* strain S12 was evaluated using strain S3 as a host.

### Mammalian cell infection assay

For cell infection experiments, mammalian cells in the logarithmic growth phase were seeded in a 96-well plate at a density of 20,000 cells per well in 100 µL of serum-free, antibiotic-free DMEM and incubated overnight at 37°C. Filtered bacterial metabolites were added to serum-free, antibiotic-free DMEM at a final concentration of 6%, with LB medium serving as a blank control. The mixtures were homogenized thoroughly before replacing the original medium in each well with 100 µL of the prepared samples. Each condition was tested in triplicate. Following a 10 h incubation at 37°C in a 5% CO_₂_ atmosphere, the medium was discarded, and 100 µL of CCK-8 reagent (Cell Counting Kit-8), diluted 10-fold in serum-free, antibiotic-free DMEM, was added to each well. After a 1 h incubation at 37°C in a 5% CO_2_ incubator, the absorbance at 450 nm (OD450) was measured using a microplate reader.

### Statistical analysis

For bacterial growth and phage production assays, statistical analyses were performed on the means of log-transformed values. Data were analyzed using GraphPad Prism 8.0 software. Unpaired *t*-tests were used for comparisons between two groups, while one-way analysis of variance followed by Dunnett’s post hoc test was used for comparisons among three or more groups. *P* values of <0.05 were considered statistically significant.

## Data Availability

Bacterial and phage accession numbers, along with the oligonucleotides used, are provided in the supplemental data. The raw data supporting the creation of all main and supplemental figures, as well as the statistical analysis conducted, are also available.

## SUPPLEMENTAL MATERIAL

Supplemental Tables and Figures msystems.00428-25-s0001.pdf Tables S1-S8; Fig. S1 to S8.

Supplemental material msystems.00428-25-s0002.xlsx The raw data and statistical analysis corresponding to each figure.

ASM does not own the copyrights to Supplemental Material that may be linked to, or accessed through, an article. The authors have granted ASM a non-exclusive, world-wide license to publish the Supplemental Material files. Please contact the corresponding author directly for reuse.
