# Supplementary material for: Systematic evaluation of phenotypic variations induced by prophages in a clinical isolate of Pseudomonas aeruginosa
Source: mSystems. 2025 Aug 15;10(9):e00428-25. doi: 10.1128/msystems.00428-25 (PMC12456005; doi:10.1128/msystems.00428-25)
Supplement: Supplemental Tables and Figures — Tables S1-S8; Fig. S1 to S8. [file msystems.00428-25-s0001.pdf]

Table S1. Summary of the 12 prophages identified in *P. aeruginosa* ZS-PA-05 by PHASTER analysis

Region	Completeness[a]	Score[b]	Length (Kb)	GC content (%)	Total Proteins	Region Position (bp)
1	incomplete	60	10.9	64.14	8	285361-296287
2-WX-3	intact	150	31	64.11	36	668942-699972
3-WX-4	intact	130	55	59.67	69	892522-947522
4	questionable	90	40.5	61.80	56	952287-992851
5-WX-2	intact	101	36.9	64.19	60	1032082-1069029
6	questionable	80	20.6	59.50	17	2901105-2921704
7	incomplete	20	10.3	55.65	10	3196970-3207312
8	questionable	70	8	61.83	9	4762604-4770605
9-WX-5	intact	130	66.9	60.93	64	5430628-5497607
10-Pf1	questionable	85	10.6	58.04	9	5601956-5612562
11-TY	incomplete	20	7.5	61.90	10	6270936-6278518
12-WX-1	intact	106	35.6	64.42	50	6345125-6380814

[a] A prediction of whether the region contains an intact or incomplete prophage based on the criteria described above.

[b]Scores of the region based on the above criteria.

Table S2. Summary of the four prophages identified in *P. aeruginosa* ZS-PA-05 by VirSorter2 analysis.

Region	Length (Kb)	Total Proteins	Region Position（bp）
1（Including WX-4）	388.6	445	890973-1279563
2（Including WX-1）	93	107	6336087-6429106
3（Including WX-5）	92.8	106	5433357-5526171
4（Including WX-3）	44.6	54	668942-713544

Table S3. Summary of the three prophages identified in *P. aeruginosa* ZS-PA-05 by CheckV analysis.

**Region**	**Length (Kb)**	**Region Position（bp）**
1（Including WX-4）	172.4	896604-1069029
2（Including WX-5）	48.7	5437025-5485694
3（Including WX-1）	37.2	6344899-6382073

Table S4. Summary of the characteristics of the eight prophages analyzed in this study

Prophage	Length (bp)	GC content (%)	Number of ORFs	Region Position （bp）
WX-1	37205	64.09	55	6345038-6382242
WX-2	37117	64.13	60	1032082-1069198
WX-3	31031	64.11	38	668942-699972
WX-4	55001	59.67	76	892522-947522
WX-5	66980	60.92	77	5430630-5497609
Y	21631	57.46	33	3659196-3680826
Pf1	10656	58.05	15	5601956-5612611
TY	10329	57.01	17	6268244-6278572

Table S5 Characterization of prophage integration sites in *P. aeruginosa* ZS-PA-05

Prophage	*attL*	*attR*
WX-1	TCCGCCACGGCGGATAACCGCACGCGGTT ATTCGCCCTACGCCCCGGTGGCCCCGACTC CCGCAGGGCGCATGACGCCAACGGCGTTA TCCGCCGACAC	TCCGCCACGGCGGATAACCGCACGCGGTT ATTCGCCCTACGCCCCGGTGGTCCCTGCTC CCGTAGGGCACATGACGCCAACGGCGTTA TCCGCCGACAC
WX-2	ND*	ND*
WX-3	ND*	ND*
WX-4	ND*	ND*
WX-5	GACGGGGGTTCAAATCCCCCCGGCTCCAC CAAATACACCAACAAA	GACGCTGGTTCAAATCCCCCCGGCTCCACC AAATGATCAAACAAA
Y	TGGCTTCGCATAATGT	TGGCTTCGCATAATGT
Pf1	AGGGTTCGATTCCCTTCGCCCGCTCCAGAT	AGGGTTCGATTCCCTTCGCCCGCTCCAGAT
TY	TGGCGGAAGGCAGTGGGAGTCGAACCC	TGGCGGAAGGCAGTGGGAGTCGAACCC

*No detection.

Table S6. Bacterial strains, cell lines, plasmids, and bacteriophages

Name	Genotype, relevant markers or source
Escherichia coli	
SM10	λ-pir， *Escherichia coli*
Pseudomonas aeruginosa	
ZS-PA-05	Wild-type, *Pseudomonas aeruginosa*, host for prophage
ZS-PA-35	Wild-type, *Pseudomonas aeruginosa*, host for phipa2 and phipa10
ZS-PA-13	This study
ZS-PA-14	This study
Δ*pilT*	In-frame deletion of *pilT* in ZS-PA-35
Δ*pilT*	In-frame deletion of *pilT* in ZS-PA-05
ΔWX-1	Deletion of prophage WX-1 in ZS-PA-05
ΔWX-2	Deletion of prophage WX-2 in ZS-PA-05
ΔWX-3	Deletion of prophage WX-3 in ZS-PA-05
ΔWX-4	Deletion of prophage WX- 4 in ZS-PA-05
ΔWX-5	Deletion of prophage WX- 5 in ZS-PA-05
ΔY	Deletion of prophage Y in ZS-PA-05
ΔPf1	Deletion of prophage Pf1 in ZS-PA-05
ΔTY	Deletion of prophage TY in ZS-PA-05
ΔALL	Deletion of all prophages in ZS-PA-05
Δ*pqsR*	In-frame deletion of *pqsR* in ZS-PA-05
Δ*trpG*	In-frame deletion of gene *trpG* in ZS-PA-05
Salmonella	
S12	Wild-type, *Salmonella* , host for prophage
S3	Wild-type, *Salmonella*
Cell	
A549	Human alveolar basal epithelial cell line
Plasmids	
pEXG2	Allelic exchange vector with pBR origin, Gm^R, *sacB*
pΔ*pilT*	Gm^R; pEXG2 derivative deleting *pilT* in-frame of ZS-PA-35
pΔ*pilT*	Gm^R; pEXG2 derivative deleting *pilT* in-frame of ZS-PA-05
pΔWX-1	Gm^R; pEXG2 derivative deleting WX-1 of ZS-PA-05
pΔWX-2	Gm^R; pEXG2 derivative deleting WX-2 of ZS-PA-05
pΔWX-3	Gm^R; pEXG2 derivative deleting WX-3 of ZS-PA-05
pΔWX-4	Gm^R; pEXG2 derivative deleting WX-4 of ZS-PA-05
pΔWX-5	Gm^R; pEXG2 derivative deleting WX-5 of ZS-PA-05
pΔY	Gm^R; pEXG2 derivative deleting Y of ZS-PA-05
pΔPf1	Gm^R; pEXG2 derivative deleting Pf1 of ZS-PA-05
pΔTY	Gm^R; pEXG2 derivative deleting TY of ZS-PA-05
*p*ΔALL	Gm^R; pEXG2 derivative deleting all prophages of ZS-PA-05
*p*Δ*pqsR*	Gm^R; pEXG2 derivative deleting *pqsR* in-frame of ZS-PA-05
pΔ*trpG*	Gm^R; pEXG2 derivative deleting *trpG*in-frame of ZS-PA-05

Bacteriophages	
phipa2	*Podoviridae*, accession number: OK539824.1
phipa3	This study
phipa4	*Siphoviridae*, accession number: OK539825.1
phipa7	This study
phipa8	This study
phipa9	This study
phipa10	*Myoviridae*, accession number: OK539826.1
phipa11	This study

Table S7.BioSample and Accession numbers about *P. aeruginosa* and prophage in this study

Name	BioSample	WGS	Accession numbers
Pseudomonas aeruginosa			
ZS-PA-05	SAMN25656732		GCA 022220245.1
ZS-PA-35	SAMN22350755	JAJDFD000000000	GCA 020567355.1
ΔWX-1	SAMN47294596	JBMBXR000000000	GCA 048627105.1
ΔWX-2	SAMN47294597	JBMBXS000000000	GCA 048627065.1
ΔWX-3	SAMN47294598	JBMBXT000000000	GCA 048627085.1
ΔWX-4	SAMN47294599	JBMBXU000000000	GCA 048628065.1
ΔWX-5	SAMN47294600	JBMBXV000000000	GCA 048628045.1
ΔY	SAMN47294603	JBMBXY000000000	GCA 048628025.1
ΔPf1	SAMN47294601	JBMBXW000000000	GCA 048628105.1
ΔTY	SAMN47294602	JBMBXX000000000	GCA 048627125.1
ΔALL	SAMN47294604	JBMBXZ000000000	GCA 048628085.1
Prophage			
Y			PV296015
Pf1			PV296013
TY			PV296014

Table S8. Oligonucleotides used in this study

Name	Source	Primers（5'-3'）
	**Prophage deletion**	
Pa269	TY_1	TTTTCTAGACCTCCCCAGCCAAACGTA
Pa270	TY_2	CAGCCGTTCCTGGGTATTCTCCGCTCGCTTTCGGCGGCCA
Pa271	TY_3	AAGCGAGCGGAGAATACCCAGGAACGGCTGCCGTCCCCCA
Pa272	TY_4	TTTAAGCTTCGCCCTTGGTGGGTAATG
YYX01	*pqsR*-1	TTTTCTAGATGACGATTGCAGGTTTCGG
YYX02	*pqsR*-2	CTGCTGGAGAACGCTCCCTTATTCCTTTTATTGGGTGGCG
YYX03	*pqsR*-3	TAAAAGGAATAAGGGAGCGTTCTCCAGCAGACGCTGGCCG
YYX04	*pqsR*-4	TTTAAGCTTCCTCGATACCAGCATCGTC
TY31	*trpG*-1	TTTTCTAGATACTCTGACTGGATCGCCA
TY32	*trpG*-2	ATTGAGGGCTCCCTTGATATCCATCGCTGGCTGTAACCTC
TY33	*trpG*-3	ATGAAATCAAGAGGTTACAGCCAGCGATGGATATCAAGGG
TY34	*trpG*-4	TTTCTCGAGGACCTGGGCGAACATGAA
	**Prophage check**	
Pa145	WX-1_1	GCCTGAACGTCAGCGCG
Pa146	WX-1_2	GGGAAGCATGCCGAGCAG
Pa161	WX-2_1	GAGCCGCCTATCGTCGCTTC
Pa162	WX-2_2	CATCCACCGCCTTCAGCGAG
Pa181	WX-3_1	GCGACTTCCATGTCGTGG
Pa182	WX-3_2	CCTGGTGCATGTGAAAGGG
Pa179	WX-4_1	CTGATCCTGCCGTCGCTG
Pa180	WX-4_2	GTGCAGGCCGGTATCAGC
Pa187	WX-5_1	CCTTGATGCTGTCGTTTCTC
Pa188	WX-5_2	CGTCATGCATGACCTTGTAG
Pa197	Y_1	GCAGGTGCATCAGCTGGCTG
Pa198	Y_2	CGTGAACGTCCGTGTCGATG
Pa203	Pf1_1	CCGCCACGTCAGCAGCCTGG
Pa204	Pf1_2	GCCGGCCTATCGTGTGGATG
Pa275	TY_1	GAAGACCGCCTCGTCGTC
Pa276	TY_2	ACCGCCTGGCCAGTCTCT
YYX09	*pqsR*-1	AAATGTGCGTCATAGTCG
YYX10	*pqsR*-2	CCATGCAGTTGCTGCGGCAG
TY37	*trpG*-1	AGTGGTTCGTCGGTTCTGT
TY38	*trpG*-2	TGGCTCTTGATCCCGAAGT
Pa77	pEXG2-1	GCAGACTACGGGCCTAAAGAAC
Pa78	pEXG2-2	CAGTAAGGCAACCCCGCCAG
	**Prophage circulation**	
TY58	WX-1_1	TGAAAGGCGAGTTCTTCGC

TY59	WX-1_2	ATGACACCTACGCCTACGT
Pa342	WX-5_1	GGTATGGACGAGATGTTGCG
Pa343	WX-5_2	CACTATCACATCCCCTGACG
TY23	Pf1_1	CGCCAGTCACTTCATCATG
TY24	Pf1_2	TCAAGGAAAACCTTGGGCA
TY27	TY_1	TATCAATTGTTCACCAGCT
TY28	TY_2	CGTGAGTTGACCTACCTG
	**Prophage-qPCR**	
TY39	WX-1_1	CTCGTCGAACTGGATGGTTT
TY40	WX-1_2	AGCTTGGAGGCAAGAAAGG
TY41	WX-2_1	CAGGGAGTGCAAGTGCTTTA
TY42	WX-2_2	GCCAAGGAAATGAGCACTCT
TY43	WX-3_1	CGGAAAGGAAACGACCTTACTC
TY44	WX-3_2	CGCCACTGTCGGGAAATAAA
TY45	WX-4_1	CAGGTCAACACGGCATAGAA
TY46	WX-4_2	CGCTACACCTTGGACATCAA
TY47	WX-5_1	CGGCTTCGAGATGGTCATTAT
TY48	WX-5_2	GAAGGTCAGAAACTCGGTAGAC
TY49	Y_1	GCTACTCGGAAGCATGTGAA
TY50	Y_2	CAAAGCCGGGCCAGTAATA
TY51	Pf1_1	GTATTGGGCGGTCTGAGTATTG
TY52	Pf1_2	CCGGCAGGTGTATTTCCTTT
TY53	TY_1	CACGAATGGAAGGCTCTGTATC
TY54	TY_2	CAGGTAGGTCTGTATCCACTCA
TY55	PSAP2-1	CGAAGGAGTCCCTGTTATTACC
TY56	PSAP2-2	CCGGCGATATGGCTACTATTC
	**Prophage upstream and downstream genes-qPCR**	
TY62	WX-1 up_1	TCACCAACTACCTGCAACC
TY63	WX-1 up_2	CCTTGGTGGAGATGCTGTT
TY64	WX-1 down_1	GGCACCACGCTCTATCTTT
TY65	WX-1 down_2	CAGACGAACAGACCCATCAG
TY66	WX-2 up_1	CCAACAGCAGTTCAGCCT
TY67	WX-2 up_2	TAGAACAGCTCGCGGTAGA
TY68	WX-2 down_1	AAAGTGGAACGGCGAGAAC
TY69	WX-2 down_2	CGACAGTTCCCAGTTGAAGG
TY70	WX-3 up_1	CGGTGATCAAGAACGGTGAA
TY71	WX-3 up_2	GCGCTTGTTGATGGTTTCTTC
TY72	WX-3 down_1	ATGGAAGTCAGCGAGGTCTA
TY73	WX-3 down_2	CACGCTCCTGGAAGAACAG
TY74	WX-4 up_1	TTCGTCGGCTTCTGGTTC
TY75	WX-4 up_2	CCGACATGGGCGATGAA

TY76	WX-4 down_1	CATTGAAGTCACCTGCGTTTC
TY77	WX-4 down_2	ATTTCCCACCGATTGCCA
TY78	WX-5 up_1	GCTTCGAGGAACTGGAGATT
TY79	WX-5 up_2	ACAGCTGGTTGACCCATT
TY80	WX-5 down_1	ATACGCCAGACATCGTCAAG
TY81	WX-5 down_2	CAGGGTAATACCTGCGAAAGA
TY82	Y up_1	GACGGAACTATAGCGACTTTGG
TY83	Y up_2	GATCCGTCGTACCGTTTCTTT
TY84	Y down_1	GGTGGAGTTCGACATCAACTAT
TY85	Y down_2	GGCGATCATGTCCTTGTCTAT
TY86	Pf1 up_1	GCATTACAACGTGGTGCTCTA
TY87	Pf1 up_2	CCTCGCATTCCTTGCTGAT
TY88	Pf1 down_1	TACCTCACCCATGAGGTCTATC
TY89	Pf1 down_2	CCGGAATAGGTTTCGTTCCA
TY90	TY up_1	GAGGAACGCCACTTCTCAG
TY91	TY up_2	GCTGGGTTGGCTGAAGT
TY92	TY down_1	GAAGAGGTGCGCTGGAT
TY93	TY down_2	CAGTGGTAAGGAAGTGGAACA

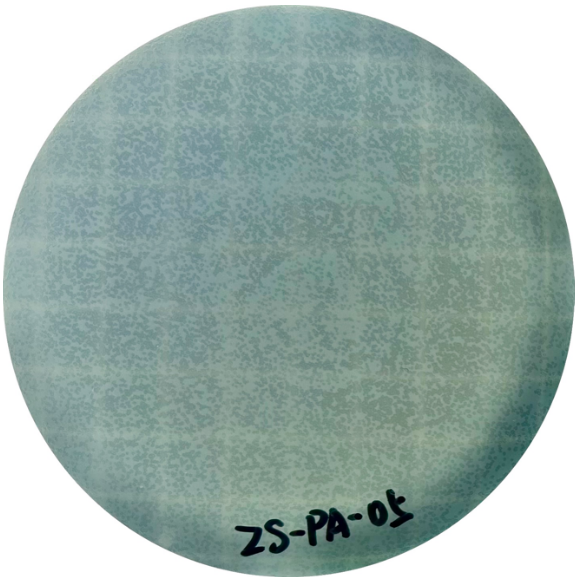

Fig. S1. *P. aeruginosa* ZS-PA-05 exhibited lysis in bacterial lawns after overnight incubation, likely due to prophage induction.

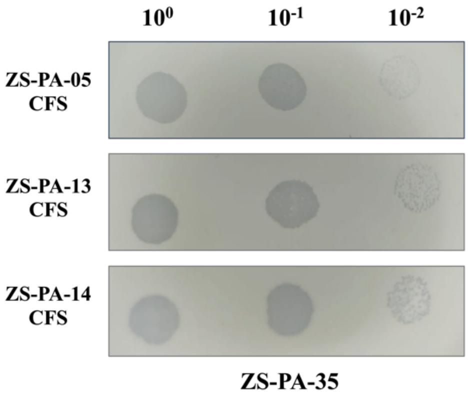

Fig. S2. The cell-free supernatant (CFS) of strains ZS-PA-05, ZS-PA-13, and ZS-PA-14 formed plaques on *P. aeruginosa* ZS-PA-35 (phage dilution gradient: 10^0, 10^{-1}, 10^{-2}).

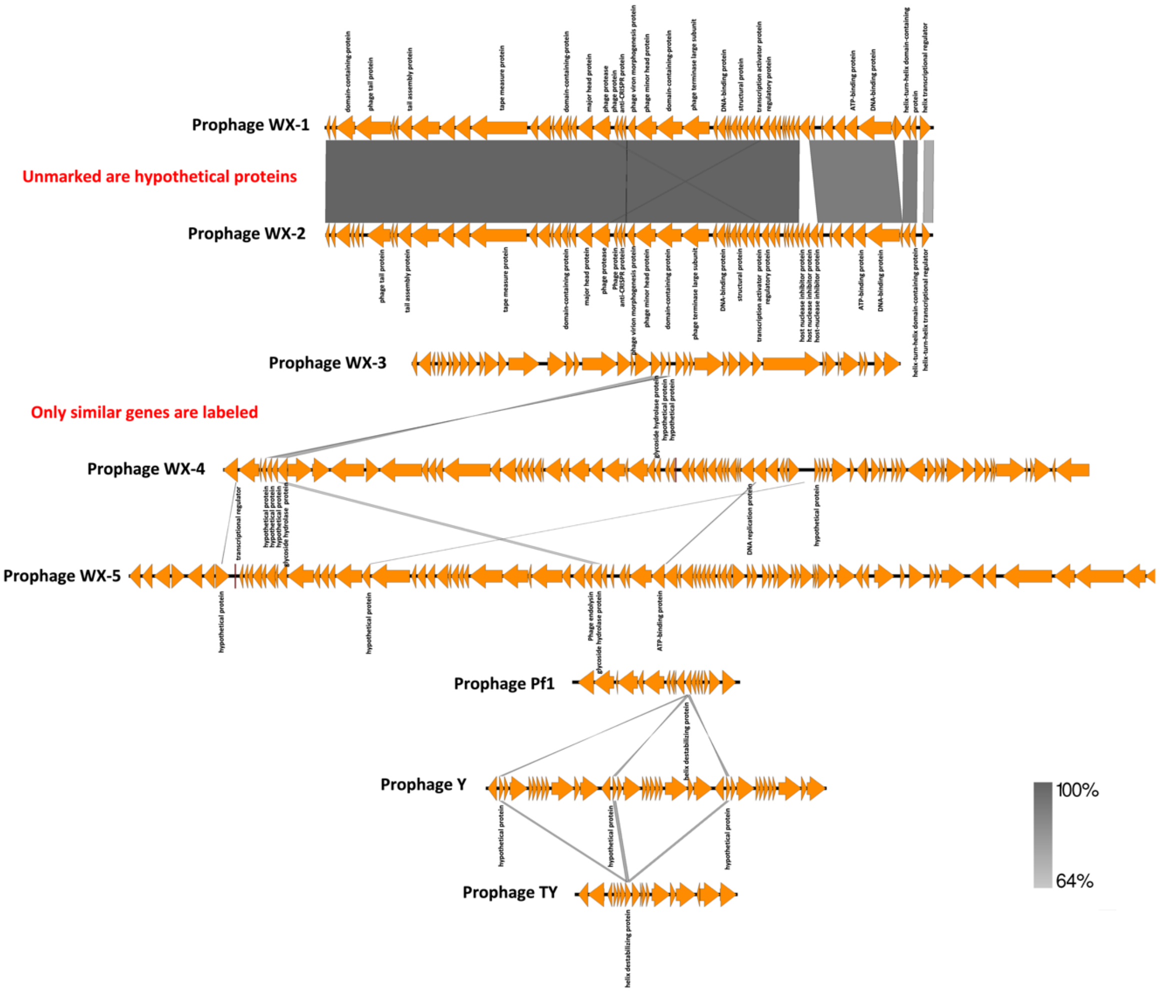

Fig. S3. Comparative analysis of the genetic similarity among the eight prophages, visualized using EasyFig, to highlight shared genomic features and regions of homology.

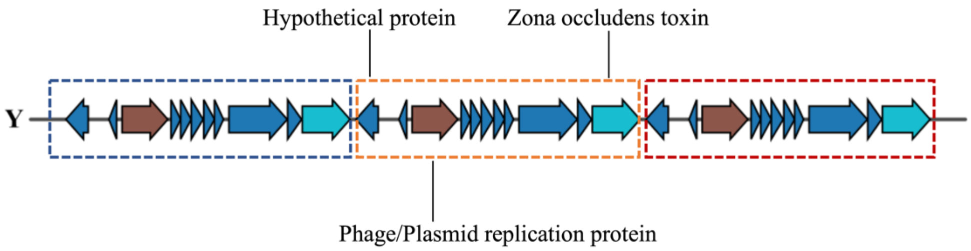

Fig. S4 Schematic diagram of the prophage Y gene sequence. Three highly repetitive gene fragments within the genome are highlighted in the dotted boxes.

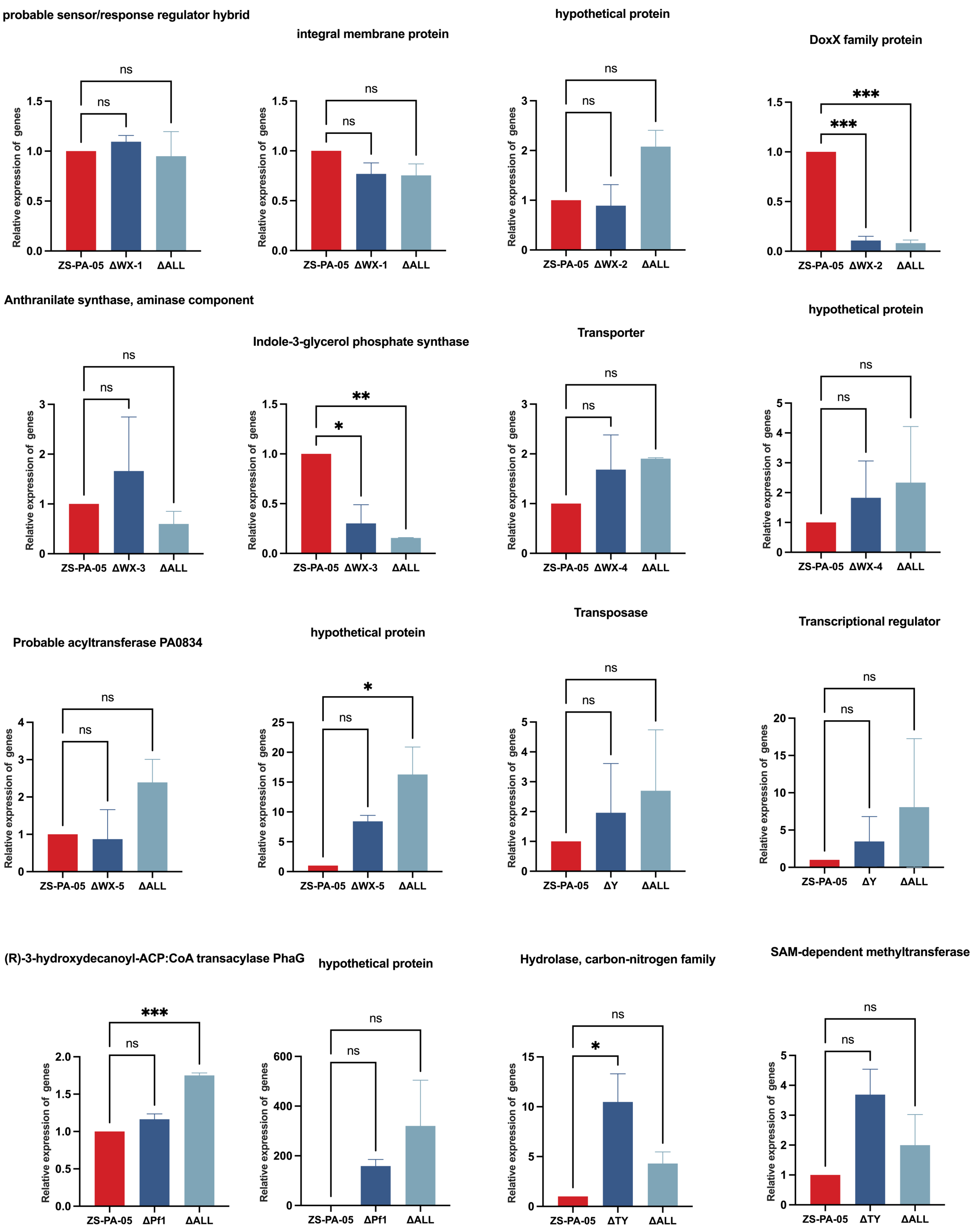

Fig. S5. Impact of prophage deletion on the transcriptional regulation of adjacent genes in *P. aeruginosa* ZS-PA-05. RT–qPCR was performed to quantify the expression levels of genes located upstream (left) and downstream (right) of the prophage integration sites. Expression in the prophage deletion mutants was compared with that in the wild-type strain, using the *rpoD* gene as an internal normalization control. The data reveal that removal of the prophages significantly alters the expression of neighboring genes, highlighting the regulatory influence of prophage elements on the bacterial genome. Statistical significance is indicated as follows: $^{*}P< 0.05$; $^{**}P< 0.01$; $^{***}P< 0.001$; $^{****}P< 0.0001$.

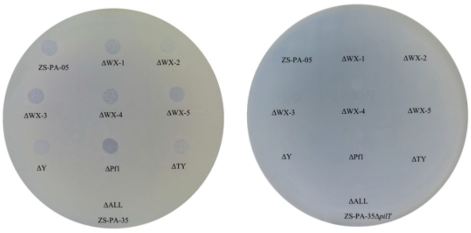

Fig. S6. Identification of prophage receptors: ZS-PA-35 and Δ*pilT* ZS-PA-35 strains were used as hosts to collect bacterial supernatants for spotting experiments.

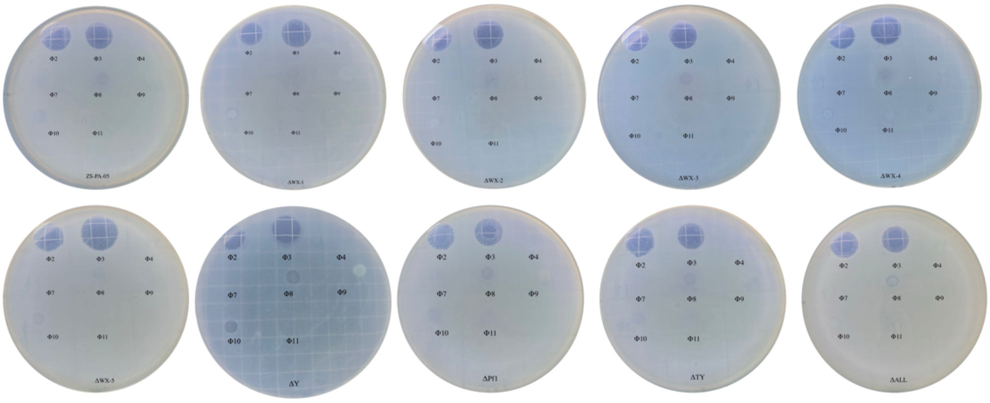

Fig S7 The effect of 8 lytic phages on wild-type strain ZS-PA-05 and its prophage knockout strain.

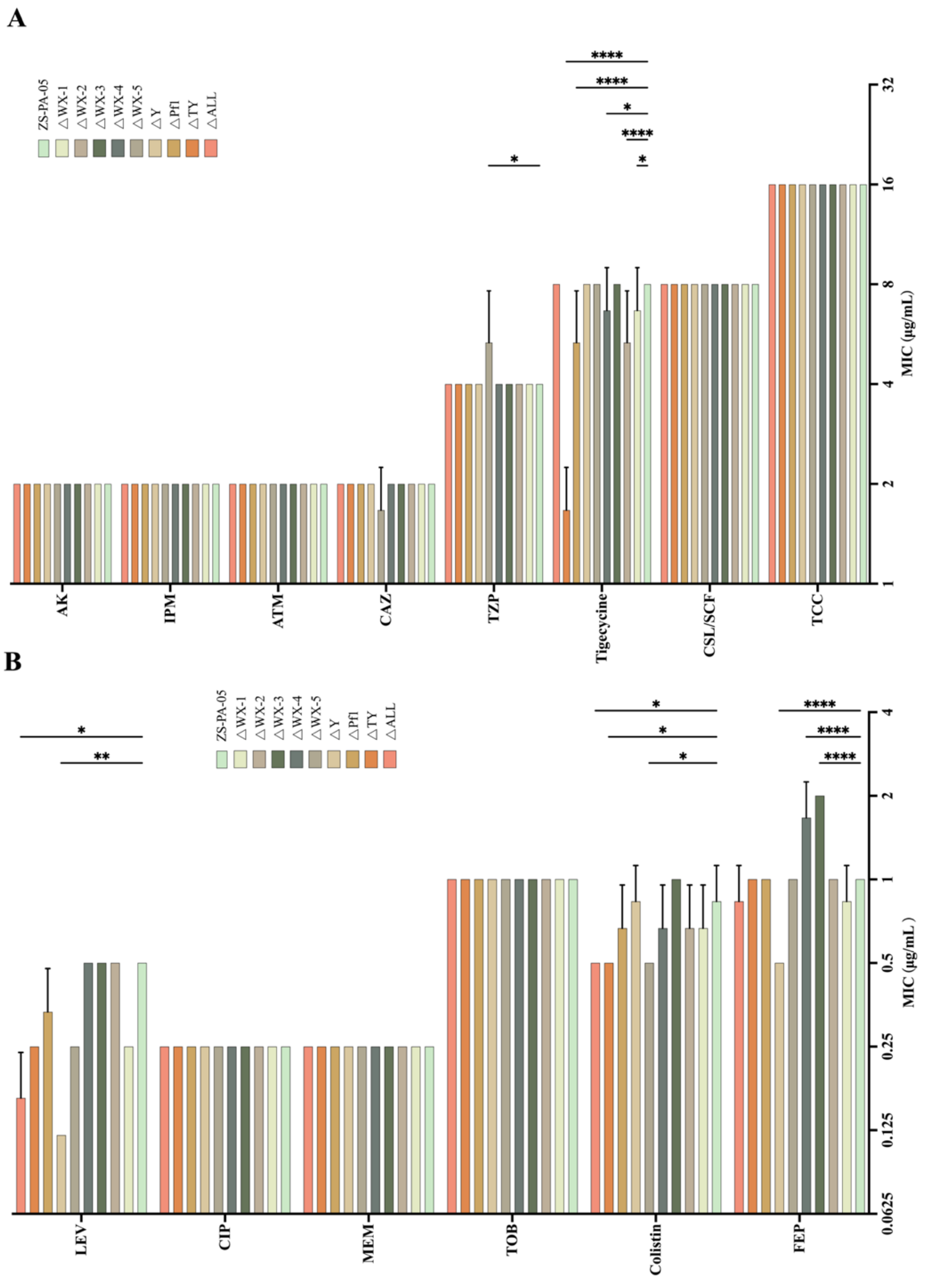

Fig S8. Effect of prophage on the antibiotic sensitivity of *P. aeruginosa* ZS-PA-05. (A, B) Antibiotic sensitivity of the wild-type strain and prophage deletion mutants was assessed using the Vitek 2 System (version 8.01). Changes in MIC values (µg/mL) of the prophage deletion strains relative to the wild-type are shown on the y-axis. Statistical significance is indicated as follows: $^{*}P< 0.05$; $^{**}P< 0.01$; $^{***}P< 0.001$; $^{****}P< 0.0001$. Error bars represent the standard deviation (n = 3). Antibiotics and antibiotic combinations are abbreviated as follows: levofloxacin (LEV), ciprofloxacin (CIP), meropenem (MEM), tobramycin (TOB), colistin, cefepime (FEP), amikacin (AK), imipenem (IPM), aztreonam (ATM), ceftazidime (CAZ), piperacillin/tazobactam (TZP), tigecycline, cefoperazone/sulbactam (CSL/SCF), and ticarcillin/clavulanic acid (TCC).
